# Early innate immune responses in European sea bass (*Dicentrarchus labrax* L.) following *Tenacibaculum maritimum* infection

**DOI:** 10.3389/fimmu.2023.1254677

**Published:** 2023-09-04

**Authors:** Inês A. Ferreira, Diogo Peixoto, Ana Paula Losada, María Isabel Quiroga, Ana do Vale, Benjamín Costas

**Affiliations:** ^1^ Abel Salazar Institute of Biomedical Sciences (ICBAS), University of Porto, Porto, Portugal; ^2^ Interdisciplinary Centre of Marine and Environmental Research (CIIMAR), University of Porto, Porto, Portugal; ^3^ Fish Immunology and Vaccinology Group, IBMC-Instituto de Biologia Molecular e Celular, Universidade do Porto, Porto, Portugal; ^4^ i3S - Instituto de Investigação e Inovação em Saúde, Universidade do Porto, Porto, Portugal; ^5^ Departamento de Anatomía, Produción Animal e Ciencias Clínicas Veterinarias, Facultade de Veterinaria, Universidade de Santiago de Compostela, Lugo, Spain

**Keywords:** tenacibaculosis, aquaculture, mucosal immunity, innate immunity, bacterial infection

## Abstract

**Introduction:**

The marine aquaculture industry has been witnessing a worldwide emergence of tenacibaculosis, a poorly understood bacterial disease caused by *Tenacibaculum maritimum* that affects commercially important fish. So far, knowledge on the *T. maritimum* virulence mechanisms is scarce and the pathogen-host interaction operating in tenacibaculosis remain to be disclosed. This study aimed at contributing to a better understanding of this disease, by evaluating the early innate immune response triggered in European sea bass (*Dicentrarchus labrax*) by a bath-challenge with *T. maritimum*.

**Methods:**

Groups of sea bass were bath-challenged with *T. maritimum* (challenged fish) or mock-challenged. Undisturbed fish were used as controls (time 0). Samples of blood, liver and mucosal organs (skin, gills and posterior-intestine) were collected at 0 h (control) and at 6, 24, 48 and 72 h post-challenge (n=12). Mucosal organs were used for analyzing the expression of immune-related genes by RT-qPCR, as well as blood samples for assessing haematological and innate humoral parameters and liver for oxidative stress assessment.

**Results:**

An increased expression of *il-1β*, *il8*, *mmp9* and *hamp1* was detected in all mucosal organs of infected fish when compared with control and mock-challenged fish, suggesting a pro-inflammatory response against *T. maritimum* transversal to all organs. The faster induction of these pro-inflammatory genes was observed in the gills. Regarding the systemic response, challenged fish presented neutrophilia, monocytosis, signs of anemia, and a decrease of bactericidal and lysozyme activities in plasma. Almost no variations were observed regarding hepatic oxidative stress.

**Discussion/Conclusions:**

The present study suggests that *T. maritimum* induces a local innate immune response upon bath infection not only in the skin of European sea bass, but also in the gills and posterior-intestine, likely triggered by the *T. maritimum*’s capacity to adhere, colonize and damage these organs that can function as entry ways to bacteria, leading ultimately to the seen host’s systemic response.

## Highlights

- Bath-challenge with *T. maritimum* induces a pro-inflammatory response in fish mucosal organs;
- The response was faster in the gills than in the skin and posterior-intestine;
- Hemato-immunological parameters of challenged fish suggest a systemic response;

## Introduction

Aquaculture is regarded as one of the fastest growing food production sectors, and, therefore, has the potential to fulfil the future demand for animal protein. This need is reflected in the tendency that aquaculture has to develop towards intensification (1), which in turn could enhance the susceptibility of the farmed aquatic organisms to disease outbreaks. The introduction and translocation of fish stocks between aquaculture facilities can also lead to the spread of diseases (2), which in association with the high stocking densities used in the aquaculture settings allow the thriving of several pathogens (3, 4).

In the last decades, the marine aquaculture sector has been witnessing a worldwide emergence of tenacibaculosis (formerly known as marine flexibacteriosis), a relatively unknown pathology that affects several commercially important species (5–7).

This disease has been responsible for countless losses, since it was first reported as a gliding bacterial infection affecting black seabream fry (*Acanthopagrus schlegeli*) reared in floating net cages in Japan (8). Since then, this pathogen was able to spread between aquaculture sites, reaching Europe in the French Mediterranean Coast, where it affected European sea bass (*Dicentrarchus labrax*) rearing facilities (9). Later on, cases of tenacibaculosis in cultured European sea bass were diagnosed in Italy, Greece and Turkey (10–12), increasing the concern regarding this disease.

*Tenacibaculum maritimum* is the etiological agent of tenacibaculosis, and has been described as a Gram-negative filamentous bacterium able to induce small lesions, upraised spots, scale loss and some disintegration of the epidermis in the host’s body surface, namely in the head, skin or fins (13–15). These lesions can establish a portal of entry for other opportunistic and frank pathogens, leading to mixed infections, which can ultimately lead to the host’s death (15–17).

In order to cause such detrimental symptomatology, *T. maritimum* presents a plethora of virulence mechanisms that allows a successful adhesion and colonization of its hosts. These bacteria rely on the production of exopolysaccharides, various adhesins and proteins with lectin or carbohydrate-binding motifs to strongly adhere to fish mucus, where they gather and accumulate the nutrients necessary for growth and proliferation (16, 18, 19). *T. maritimum* has also been described as a pathogen able to agglutinate erythrocytes from a wide range of species (20) and to directly compete with the host’s iron-binding proteins. In a study developed by (21), it was demonstrated that different *T. maritimum* strains have at least two different iron-uptake mechanisms, one related to the synthesis of siderophores and other involving the utilization of heme groups as iron sources (21). The proteolytic activity of several extracellular products (ECPs) has also been described and shown to include the ability to degrade gelatin, amylase, casein and nucleases (20). Furthermore, the genome analysis of *T. maritimum* revealed several proteins homologous to proteins that in other bacteria are known to act as toxins and virulence factors, such as sphingomyelinase and ceramidase (19). Despite these studies, knowledge regarding *T. maritimum* pathogenesis is scarce, and very few studies have approached the interactions between this pathogen and the host.

Guardiola et al. (22) focused on Senegalese sole (*Solea senegalensis*) mucosal and systemic immune responses following bath challenge with a sub-lethal dose of *T. maritimum* and further suggested the rudimentary systemic and the delayed host’s mucosal responses (22). Plasma’s antiprotease and bactericidal activities were mainly increased in challenged fish in the end of the trial, at 14-days post-challenge, and the same tendency was recorded for the haemolytic complement, lysozyme and peroxidase activities in skin mucus (22). This suggests that Senegalese sole immune response can be prolonged at least 14 days after being exposed to *T. maritimum*.

In a study developed by Faílde et al. (23), the haematological profile of turbot (*Scophthalmus maximus*) challenged subcutaneously with *T. maritimum* showed some alterations, including granulocytosis, lymphopenia and thrombocytopenia as well as mild decrease of haematocrit values. Due to the seen distribution of immunoglobulin positive cells in spleen, kidney, thymus, skin and intestine, it is suggested that tenacibaculosis is able to induce a humoral immune response in turbot, through the synthesis of specific antibodies in the spleen that later on migrate to lesion areas in the skin (23).

The present study aimed to bring more insights on the host responses against this fastidious bacterial pathogen by evaluating parameters of the short-term mucosal and systemic innate immune response in European sea bass (*Dicentrarchus labrax*) after bath-challenge with *T. maritimum*. To the best our knowledge, this is the first study approaching the host’s molecular immune response with focus on the three main mucosal organs (gills, skin and posterior-intestine).

## Material and methods

### Bacterial culture and inoculum preparation

The *T. maritimum* strain (ACC13.1) used in this study was isolated from Senegalese sole and belongs to the serotype O3 (24). The strain was kindly provided by Professor Alicia E. Toranzo (Departamento de Microbiología y Parasitología, Facultad de Biología, University of Santiago de Compostela, Spain) and stocks were kept frozen at −80°C until use. Recovery from frozen stocks was achieved using marine agar (MA; Laboratories CONDA, Spain) at 25°C for 48 h.

For inoculum preparation, bacteria were inoculated in 50 mL of marine broth (MB; Laboratories CONDA, Spain) in a 500 mL Erlenmeyer and grown at 25°C, with continuous shaking (180 rpm) for 48 h. Turbidity was measured at 600 nm (Spectrophotometer, UV-1600PC, VWR) and exponentially growing bacteria (OD=0.886) were collected by centrifugation at 3,000 *x g* for 10 min and resuspended in MB at a concentration of 5 x 10^5^ CFU mL^-1^. The bacterial concentration was adjusted with the predetermined growth curve for this specific strain: y = 2 x 10^8^x + 4 x 10^7^ (25).

### Fish husbandry and experimental design

The current study was conducted under the supervision of accredited researchers in laboratory animal science by the Portuguese Veterinary Authority following FELASA category C recommendations and in agreement with the guidelines for protection of animal used for scientific purposes according to European Union directive (2010/63/EU) (reviewed and approved by 0421/000/000/2020).

For this trial, European sea bass juveniles (45.45 ± 8.1 g) with no record of previous tenacibaculosis outbreaks were obtained from a commercial fish farm (Portugal) and were maintained in quarantine for 4 weeks at CIIMAR fish holding facilities in a recirculating aerated seawater system at 21.8 ± 0.4°C, salinity of 34.2 ± 0.4 ‰, 8.2 ± 0.2 mg mL^-1^ dissolved oxygen and a 12 h light/12 h dark photoperiod. Water quality was maintained with mechanical and biological filtration, and fish were fed daily with a commercial diet (Aquasoja, Portugal) at 2% of body weight, distributed by two meals a day. Ammonia and nitrite levels were measured daily using commercial kits. For screening purposes and to assess the health status of the stock fish, ten randomly selected individuals were sampled for histopathological assessment. Before the bacterial challenge, fish were randomly distributed into two closed recirculating seawater systems (10 kg m^-3^ stocking density, n= 25 fish *per* tank, 0.11 m^3^), one for the mock-challenged fish and another for the challenged fish, each with four aquaria (4 replicates for each treatment) for sampling purposes and two aquaria (two replicates for each treatment) to follow cumulative mortality, and acclimated for one week.

At the challenge, water temperature was increased to 25°C, to mimic temperature conditions at which tenacibaculosis outbreaks occur (25, 26). Fish, previously fasted for 24 h, were bath challenged for 2 h with *T. maritimum* (ACC13.1), prepared as described in the previous section (25), at a concentration of 5 x 10^5^ CFU mL^-1^ (according to a pre-challenge to determine the LD_30_ for this strain). Challenge was performed in 50 L tanks with strong aeration at a stocking density of 25 kg m^-3^. Mock-challenged fish were submitted to the same treatment, but MB was used instead of bacterial inoculum. After challenge, fish were returned to the recirculating system where they were acclimated. Bacteria was re-isolated from aseptically collected blood from randomly selected challenge fish at 24 h post-challenged and identified as *T. maritimum* as described elsewhere (27).

### Sampling

Fish were not fed during the trial period. Samples were collected post-mortem after euthanizing the fish with an overdose of anaesthetic, 0.7 mL/L (2-phenoxyethanol; Merck, ref. 807291, Germany). Sampling was performed before starting the bath challenge (n=12) (time 0, control) and at 6, 24, 48 and 72 h post-challenge. At each sampling time, three fish were removed from each tank (n=12 per treatment) and blood was collected from the caudal vein with heparinized 1 mL syringes and placed in heparinized 1.5 mL tubes. An aliquot was removed for haematological analysis, while the remaining blood was centrifuged for 10 min at 10,000 *x g* at 4°C for plasma collection and storage at ‐80°C. Skin (collected across the midline of the fish, beneath the dorsal fin, without any muscle), gills (portion of the second arch) and posterior intestine were also sampled and stored in RNA later (at a proportion of 1/10 w/v) at 4 °C for the first 24 h, and then stored at -80 °C for molecular biology analysis. Liver was collected and immediately frozen in liquid nitrogen, followed by storage at -80 °C. Samples of skin (across the midline of the fish, beneath the dorsal fin, not previously sampled for mucus, including 1 cm of subjacent muscle), gills (portion of the second arch) and posterior intestine were also collected for histological analyses.

### Haematological parameters

The haematological profile was conducted according to Machado et al. (28). Total white (WBC) and red (RBC) blood cells were counted using a Neubauer chamber and haematocrit (Ht) and haemoglobin (Hb; SPINREACT kit, ref. 1001230, Spain) were also assessed, as previously described (28). The mean corpuscular volume (MCV), mean corpuscular haemoglobin (MCH) and mean corpuscular haemoglobin concentration (MCHC) were calculated (28).

Blood smears were done with 3 µL of gently homogenized blood, air dried and fixed for 1 min in formol-ethanol (10% of 37% formaldehyde in absolute ethanol). For identifying neutrophils, the peroxidase detection method described by Afonso et al. (29) was used (29). Blood smears were then stained with Wright’s stain (Haemacolor; Merck). Slides were examined under oil immersion (1,000 x) and 200 leucocytes were counted and categorized, based on their morphology, as thrombocytes, lymphocytes, monocytes and neutrophils. The percentage of each cell population was calculated and multiplied by total number of WBC in order to determine the number of cells per mL.

### Innate immune parameters

#### Antiprotease and protease activities

The antiprotease activity was determined as described by Ellis (30) adapted for 96-well microplates. Shortly, 10 µL of plasma were incubated in microtubes with 10 µL of trypsin solution (5 mg mL^−1^ in 0.5% NaHCO_3_, pH 8.3) (Sigma, USA) for 10 min at 22°C. After incubation, 100 µL of phosphate buffer (115 mM NaH_2_PO_4_, pH 7.0) plus 125 µL of azocasein (20 mg mL^−1^ in 0.5% NaHCO_3_, pH 8.3) were added and incubated again for 1 h at 22°C in the dark, with agitation. Then, 250 µL of 10% cold trichloroacetic acid (TCA) were added and incubated for 30 min at 22°C, followed by centrifugation at 10,000 *x g* for 5 min at room temperature (RT). Finally, 100 µL were transferred, in duplicate, to a 96-well plate containing 100 µL of 1N NaOH per well and the OD (optical density) read at 450 nm in a Synergy HT microplate reader. Phosphate buffered saline was used as positive control, instead of plasma, and the percentage of trypsin activity was calculated as follows: 100 − ((sample absorbance/reference absorbance) × 100).

To assess protease activity, the same protocol was followed, but the initial incubation of the plasma with trypsin was omitted and the incubation with azocasein and phosphate buffer was maintained for 24 h instead of 1h, in constant agitation. Plasma was replaced by trypsin (5mg ml^−1^, Sigma) as a positive control or by PBS as negative control. The percentage of trypsin activity compared to the positive control was calculated as follows: (sample absorbance/positive reference) × 100.

#### Peroxidase

Peroxidase activity was determined in plasma as described by Quade and Roth (31). Briefly, in triplicates, 15 µL of plasma were placed into flat-bottomed 96-well plates and diluted in 135 µL of HBSS without Ca^+2^ and Mg^+2^ (Cytiva, USA). Then, 50 µL of 20 mM 3,3’,5,5’- tetramethylbenzidine hydrochloride (TMB; Sigma, USA) were added to each well. After 2 min the reaction was stopped by adding 50 µL of 2 M sulphuric acid and the absorbance was measured at 450 nm (Synergy HT microplate reader). Peroxidase activity (units mL^-1^ plasma) was calculated by defining one unit of peroxidase as the amount needed to produce an absorbance change of 1 OD.

#### Lysozyme activity

Lysozyme activity was assessed as described by Costas et al. (32). Firstly, *Micrococcus lysodeikticus* solution (0.5 mg mL^−1^ in 0.05 M sodium phosphate buffer, pH 6.2) was prepared. Then, 15 µL of plasma were added, in triplicates, to a microplate plus 250 µL of the *Micrococcus lysodeikticus* solution, for a final volume of 265 µL. After incubation at 25°C, the absorbance (450 nm) was measured after 0.5 and 20 min in a Synergy HT microplate reader. Lyophilized hen egg white lysozyme (Sigma) was successively diluted in sodium phosphate buffer (0.05 M, pH 6.2) to obtain a standard curve. The amount of lysozyme in the sample was calculated using the standard curve.

#### Bactericidal activity

The bactericidal activity assay was performed using *T. maritimum* ACC13.1 strain. Bacteria were grown on MA at 25°C for 24 h and resuspended in MB at a concentration of 1.6 x 10^8^ CFUs mL^-1^, by measuring the turbidity at 600 nm (Synergy HT microplate reader) and using the previously mentioned growth curve. Plasma bactericidal activity was then determined following the method described by Graham et al. (33) with some modifications (28, 33). In a U-shaped 96-well plate, 20 µL of plasma were added in duplicates, and as positive control, MB was added to the wells instead of plasma. In each well, 20 µL of bacteria were added to the plate followed by an incubation for 2.5 h at 25°C. Afterwards, 25 µL of 3-(4, 5 dimethyl-2-yl)-2,5-diphenyltetrazolium bromide (MTT, 1 mg mL^−1^; Sigma) were added to each well and the plate was incubated for 10 min at 25°C. Plates were centrifuged at 2,000 *× g* for 10 min and formazan precipitate was dissolved with 200 µL of dimethyl sulfoxide (Sigma). The absorbance of the dissolved precipitate was measured at 560 nm (Synergy HT microplate reader). In this method, the difference between the formazan present in samples and in the positive controls (100%) enables to calculate the viable bacteria in each sample and, consequently, the percentage of non-viable bacteria.

#### Nitrite concentration

To indirectly access the nitric oxide (NO) concentration in plasma, a Nitrite/Nitrate colorimetric kit (Roche, 11746081001, Germany) was used according to the manufacturer’s instructions. Since nitrite and nitrate are endogenously produced as oxidative metabolites of the messenger molecule NO, these compounds are considered as indicative of NO production (34). To measure nitrite/nitrate, the samples were previously diluted 1:10 in distilled H_2_O in microtubes and the concentrations were expressed as µM.

### Oxidative stress biomarkers

Liver tissue were homogenized 1/10 (w/v) in potassium phosphate buffer (0.2 M, pH 7.4). From the homogenized mixture, 200 µL were transferred to a microtube with 4 µL of 4% BHT (2,6-Di-tert-butyl-4-methylphenol) in methanol for lipid peroxidation (LPO) assessment.

For determining superoxide dismutase, catalase and glutathione-S-transferase activities, for each volume of tissue homogenate, a volume of potassium phosphate buffer (0.2 M, pH 7.4) was added followed by a centrifugation at 10,000 *x g* for 20 minutes at 4°C. The supernatants were collected and kept at −80°C. Protein concentration was measured using Pierce™ BCA Protein Assay kit, with bovine serum albumin as standard, according to the manufacturer’s instructions. For superoxide dismutase and catalase activity homogenates were diluted to achieve a final protein concentration of 0.3 and for total glutathione-S-transferase a concentration of 0.7 mg/mL.

LPO was determined using the protocol described by Bird and Draper (35) with some modifications (36). A volume of 100 µL of 100% TCA was added to the previously mentioned 204 µL of liver homogenate, and afterwards, 1 mL of 0.73% thiobarbituric acid solution (in Tris–HCl 60 mM, pH 7.4 with DTPA 0.1 mM). Samples were incubated for 1 h at 100°C in a kiln and then microtubes were centrifuged for 5 minutes at 15,000 *x g*. A volume of 200 µL of supernatant was transferred to a 96-well plate in triplicates and the absorbance was measured at 535 nm. The LPO was expressed as nmol of thiobarbituric acid reactive substances (TBARS) formed *per* g of wet tissue.

Catalase activity was quantified measuring the decrease in absorbance, through the consumption of H_2_O_2_, as described by Claiborne (37) but adapting the protocol to microplates as described by Rodrigues et al. (38). A sample of 10 µL was transferred to a UV light microplate in triplicates with 140 µL of potassium phosphate (0.05 M, pH 7.0) plus 150 µL of 30% H_2_O_2_. The absorbance was measured at 240 nm for 2 min. The catalase activity was quantified using H_2_O_2_ molar extinction coefficient at 240 nm of 40 M cm^-1^, expressed in U *per* mg of protein.

Superoxide dismutase (SOD) activity was assessed following the protocol describe by Almeida et al. (39), utilizing the cytochrome C method with xanthine/xanthine oxidase (39). A volume of 50 µL of each sample was transferred to a microplate in triplicates. Then, 200 µL of a reaction solution containing 50 mM potassium phosphate buffer (pH 7.8) containing 1 mM Na-EDTA, 0.7 mM xanthine and 0.03 mM cytochrome C were added. Promptly, 50 µL of 0.03 U mL^-1^ xanthine oxidase with 0.1 mM Na-EDTA were also added to the microplate. Absorbance was measured at 550 nm (Synergy HT microplate reader) at 20 s intervals for 3 min. Activity is described as units of SOD *per* mg of protein. One unit of activity was defined as the quantity of enzyme necessary to produce a 50% inhibition of the cytochrome C reduction rate.

Glutathione-S-transferase (GST) activity was accessed following the method of (40) adapted to microplate by Frasco and Guilhermino (41). Briefly, a 250 µL of a reaction solution containing 0.2 M potassium phosphate buffer (pH 6.5), 10 mM reduced glutathione (GSH) and 60 mM 1-chloro-2,4-dinitrobenzene (CDNB) was added to 50 µL of liver homogenate in triplicates. Absorbance was recorded at 340 nm for 5 min with 20 s intervals in microplate. GST activity was expressed as mU per mg of protein, using the molar extinction coefficient at 340 nm of 9.6 × 10^6^ M/cm.

The reduced (GSH):oxidized (GSSG) glutathione ratio was determined using the microplate assay for GSH/GSSG commercial kit (Oxford Biomedical Research, UK) as previously described by Hamre et al. (42). This method relies on the quantitative determination at 412 nm of the total amount of glutathione (GSH + GSSG) and GSSG (43). Briefly, the determination of GSSG is obtained by adding a thiol scavenger (N-ethylmaleimide pyridine derivative solution, Oxford Biomedical Research, UK), which reacts with GSH to form a stable complex, therefore removing the GSH prior to the quantification of GSSG, without inhibiting GR activity. Through the addition of glutathione reductase, the available GSSG is reduced to GSH which reacts with 5,5’-dithiobis-2-nitrobenzoic acid (DTNB) allowing the measurement of pre-existent GSSG. The rate of the reaction is proportional to the GSH and GSSG concentration. The GSH/GSSG Ratio is calculated as follows: (GSH_t_ – 2GSSG)/GSSG.

### Histology and immunohistochemistry

At each sampling point, 12 fish *per* group (control, mock-challenged and challenged) were sampled. Tissue fragments from gills, skin and intestine were fixed with 4% buffered formaldehyde for 24-48 h, dehydrated and embedded in paraffin wax. Sections of 2-3 mm thickness were obtained and collected on silane coated slides, followed by drying overnight, dewaxing, hydration. Sections were then stained with haematoxylin and eosin (H&E) or used for immunohistochemistry (IHC). Regarding IHC, incubations were performed at RT in a humidified chamber and washing was performed by immersion for 5 min in phosphate-buffered saline (PBS; 8 mM Na_2_HPO_4_ 3 mM NaH_2_PO_4_, 150 mM NaCl, pH 7.4) containing 0.5% (v/v) Tween 20. Endogenous peroxidase activity was quenched by incubation with peroxidase blocking buffer (Vector Labs, Burlingame, CA) for 1 h. The sections were washed once and blocked for 20 min in 2.5% normal horse serum (Vector Labs, Burlingame, CA), followed by incubation with 1:1000 (concentrations of mg mL-1) working dilution of rabbit anti-*T. maritimum* LL01.8.3.8 immunoadsorbed antibody (anti-Tm) for 1.5 h, according to Faílde et al. (23).

After washing again, the sections were incubated with ImmPRESS^®^-VR Horse Anti-Rabbit IgG Polymer-HRP (Vector Labs, Burlingame, CA) for 30 min, rinsed, and color development achieved with Vector^®^ VIP Substrate Kit, Peroxidase (HRP) (Vector Labs, Burlingame, CA), as the chromogen. After a final wash, the slides were counterstained with haematoxylin, dehydrated and mounted.

### Gene expression analysis

Target organs (gills, skin and posterior-intestine) were weighted (up to 300 mg of organ), placed in 500 µL of Trizol (NZYTech, Lisbon, Portugal) and homogenized in a Precellys Evolution homogenizer at 6000 *x g* (2 x 20 s, 4°C). After this step, 150 µL of chloroform were added at 4°C and the samples were vortexed, followed by a centrifugation at 12,000 *x g* for 15 min at 4°C. The aqueous phase was transferred to a clean tube with 300 µL of 70% ethanol, mixed, and placed in NZYSpin Binding columns. After this step, the total RNA isolation was conducted with NZY Total RNA Isolation kit (NZYTech, Lisbon, Portugal) according to the manufacturer’s specifications. RNA samples were quantified and purity was assessed by spectrophotometry using DeNovix DS-11 FX (Wilmington, DE, USA) with absorbance ratios at 260 nm/280nm of 1.9–2.1. First-strand cDNA was synthesized and samples were standardized with NZY First-Strand cDNA Synthesis Kit (NZYTech, Lisbon, Portugal) with further storage at -80°C. For reverse transcriptase, a Veriti DX 96-well Thermal Cycler (Applied Biosystems, Foster City, CA, USA) was used. Real-time Quantitative PCR (qPCR) was performed with CFX384 Touch Real-Time PCR Detection System (Biorad, Hercules, CA, USA) using 4.4 µL of diluted cDNA mixed with 5 µL of iTaq Universal SYBR Green Supermix^®^ (Biorad, Hercules, CA, USA) and 0.3 µL (10 µM) of each primer in a final volume of 10 µL. Primers were designed with NCBI Primer Blast Tool and IDT OligoAnalyzer ToolTM to amplify genes related with innate immune response in European sea bass. The known qPCR requirements (amplicon size, Tm difference between primers, GC content, and self-dimer or cross dimer formation) were respected. The template sequences used for the primer’s design were obtained from both NCBI and the databases dicLab v1.0c sea bass genome (44). The efficiency of each primer pair was determined by calculating the slope of the regression line of the cycle thresholds (Ct) vs. the relative concentration of cDNA, using serial 2-fold dilutions of cDNA. In order to ensure no amplification of primer dimers, melting curves were analyzed. The standard cycling conditions were 95 °C initial denaturation for 10 min, followed by 40 cycles of two steps (95 °C denaturation for 15 s followed by primer annealing temperature for 1 min), 95 °C for 1 min followed by 35 s at the annealing temperature, and finally, 95 °C for 15 s. The reactions were run in duplicates and target gene expression was normalized using the geometric mean of elongation factor 1β (*ef1β*) and ribosome 40s subunit (*40s*) and calculated according to the Pfaffl method (45).

Accession numbers, primer efficiencies and annealing temperatures for each organ, amplicon length and primer sequences are detailed in **Table 1**.

**Table 1:** Immune-related genes analyzed by Real-time PCR.

Gene	Acron.^a^	Accession number	Eff^b^			AT^c^ (°C)			Amplicon length	Primer sequence (5’-3’)
			Gills	Skin	PI^c^	Gills	Skin	PI^d^		
Elongation factor 1-beta	*ef1b*	AJ866727.1	114.3	96.1	107.2	60	60	60	144	F: AACTTCAACGCCCAGGTCAT R: CTTCTTGCCAGAACGACGGT
40s Ribosomal protein	*40s*	HE978789.1	108.2	96.1	105.2	60	60	62	79	F: TGATTGTGACAGACCCTCGTG R: CACAGAGCAATGGTGGGGAT
Interleukin 1-beta	*il-1β*	AJ269472.1	93.1	93.4	114.4	60	60	60	105	F: AGCGACATGGTGCGATTTCT R: CTCCTCTGCTGTGCTGATGT
Interleukin 8	*il-8*	AM490063.1	93.1	90.6	100.1	60	60	60	140	F: CGCTGCATCCAAACAGAGAGCAAAC R: TCGGGGTCCAGGCAAACCTCTT
Interleukin 6	*il-6*	AM490062.1	89.6	86.9	101.8	60	60	62	81	F: AGGCACAGAGAACACGTCAAA R: AAAAGGGTCAGGGCTGTCG
Tumor necrosis factor alpha	*tnfα*	DQ070246.1	104.9	91.3	110.7	60	55	55	112	F: AGCCACAGGATCTGGAGCTA R: GTCCGCTTCTGTAGCTGTCC
Interleukin 10	*il-10*	AM268529.1	114.9	87.3	105.5	60	60	60	164	F: ACCCCGTTCGCTTGCCA R: CATCTGGTGACATCACTC
Matrix metallopeptidase 9	*mmp9*	FN908863.1	104.5	90.5	107.8	57	62	60	166	F: TGTGCCACCACAGACAACTT R: TTCCATCTCCACGTCCCTCA
Hepcidin	*hamp1*	KJ890396.1	110.2	92.5	110.2	62	62	60	148	F: ACACTCGTGCTCGCCTTTAT R: TGTGATTTGGCATCATCCACG
Ferroportin	*fpn1*	KU599935.1	97.6	95.3	99.5	60	60	60	161	F: GCTAGAGTTGGCCTGTGGTC R: GGGTTCGGAGCCAGTATCAC
Nuclear factor kappa B	*nf-κB*	DLAgn_00239840^e^	113.3	106.8	106.8	60	60	55	136	F: GCTGCGAGAAGAGAGGAAGA R: GGTGAACTTTAACCGGACGA
Signal transducer and activator of transcription 3	*stat3*	DLAgn_00192560^e^	97.6	104.4	112.8	60	60	60	275	F: GACATCAGCGGAAAGACCCA R: GGGGTGACGCAGATGAACTT
Apoptosis regulator bcl-2-like	*bcl2-like*	DLAgn_00005980^e^	101.3	88.6	106.8	60	62	62	181	F: CTCCTCCTCCTCTTCCTCGT R: TCATCTGGTTGCTTCAGTCG
*Nod-like receptor 1*	*nod1*	DLAgn_00065300^e^	92.4	94.5	107.2	60	60	60	293	F: ACCCAAGCAATGACGTAGCA R: TTTTCCTACACCCGCATCCC
*Nod-like receptor 2*	*nod2*	DLAgn_00155640^e^	103.9	90.6	104.4	60	60	60	298	F: GAGGAAGCATCACAGGGACC R: TGCAATCCCCTCAAAGGCAA
*Toll-like receptor 2*	*tlr2*	KX399288.1	106.5	96.1	97.6	57	60	60	173	F: CAGTAGGCCAAGTCCGTCTC R: GGAGCTACGCTTGGCCTTTA
*Toll-like receptor 9*	*tlr9*	KX399289	104.5	102.7	108.2	60	55	55	100	F: TCTTGGTTTGCCGACTTCTTGCGT R: TACTGTTGCCCTGTTGGGACTCTGG

a Gene acronym.

b Efficiency of PCR reactions, calculated from serial dilutions of organ RT reactions in the validation procedure.

c Annealing temperature for each organ (°C).

d Posterior intestine.

e Sequences obtained from databases dicLab v1.0c sea bass genome.

### Statistical analysis

Mean and standard error of the mean (mean ± SEM) were calculated for all parameters. Data were analyzed for normality and homogeneity of variance, when necessary outliers were removed and gene expression data was Log-transformed before being statistically analyzed.

When all the assumptions were fulfilled, a T-student test or a One-Way ANOVA (Tukey *post hoc* test) was used under SPSS 27 program for WINDOWS. When the assumptions were not verified a Welch ANOVA (Games-Howell *post hoc* test) or a Kruskal-Wallis was performed. The level of significance used for all statistical tests was p ≤ 0.05.

## Results

### Bacterial challenge

**Figure 1:**
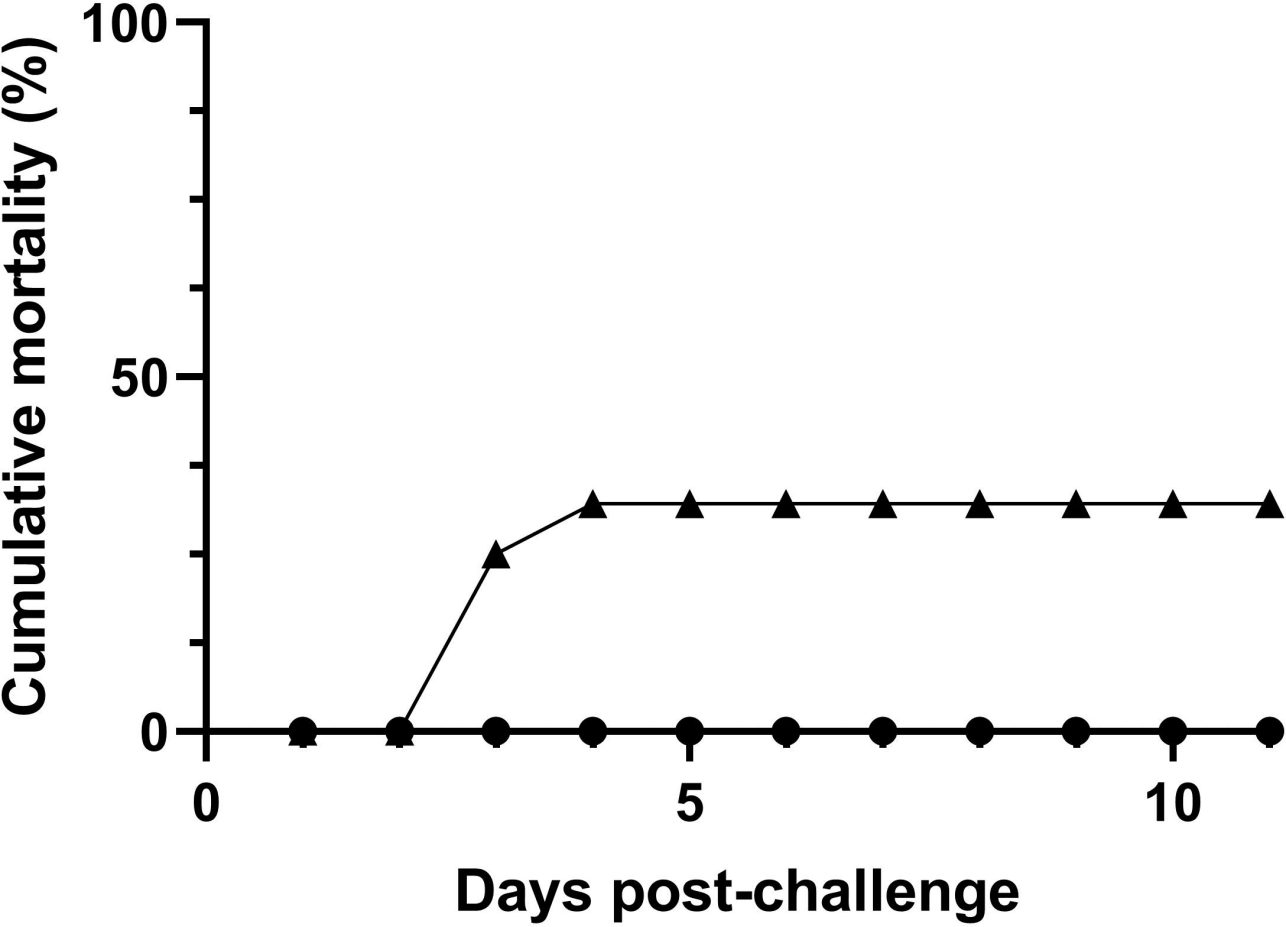
Mortality of European sea bass (*Dicentrarchus labrax*) after bath-challenge with 5 x 10^5^ CFU mL^-1^ *T. maritimum* (▲) or with marine broth MB (●) (n=30 fish per treatment).

Bath-challenge with 5 x 10^5^ CFU mL^-1^ *T. maritimum* ACC13.1 resulted in 32.1% cumulative mortality, whereas, as expected, no mortality was recorded in mock-challenged fish (**Figure 1**; n=30 fish per treatment, X^2^<0.0008 for comparisons between treatments). Moreover, the mortalities in challenged fish occurred between days 3 and 4 after challenge (**Figure 1**).

### Haematological analysis

The concentration of red blood cells suffered a decrease in infected fish at 6, 24 and 48 h post-challenge, returning to a value similar to the control fish (0 h) at 72 h (**Supplementary Table 1**). In contrast, in mock-challenged fish, a slight decrease in red blood cells was only observed at 48 h post-challenge. Furthermore, the concentrations of red blood cells at 6, 24 and 48 h in challenged fish were lower than in mock-challenged animals (**Supplementary Table 1**). In agreement with this, haematocrit also decreased at 24 and 48 h post-challenged in infected fish, when compared to control value (0 h), and was lower in infected fish than in mock-challenged fish at all-time points analyzed (**Supplementary Table 1**). However, haemoglobin did not show any significant difference for the challenged fish (**Supplementary Table 1**). The mean corpuscular volume was increased at 6 h in infected fish, when compared to controls (0h) and to mock-challenged fish (**Supplementary Table 1**), whereas for mean corpuscular haemoglobin, an increase was observed in infected fish from 6 to 48 h post-challenge when compared to controls and mock-challenged animals (**Supplementary Table 1**). Regarding mean corpuscular haemoglobin concentration, an increase at 24 and 48 h post-challenge was observed in infected fish, but the values for the remaining time points were similar between mock and challenged fish (**Supplementary Table 1**).

The white blood cells’ counts in infected fish at 6, 24 and 48 h did not differ from the control value (0 h), but at 72 h, an increase in white blood cells number was observed (**Supplementary Table 1**). Likewise, an increase in white blood cells at 72 h was registered in the mock-challenged group (**Supplementary Table 1**). The differential counts showed that the number of circulating neutrophils increased at 6, 48 and 72 h post-challenge in infected fish, compared to controls (**Supplementary Table 2**). Infected fish also presented monocytosis at 48 and 72 h, compared to controls and mock-challenged fish (**Supplementary Table 2**). Lymphocytes’ concentration did not significant differences between groups (despite a wave-like variation on its values), except at 72 h, where an increase was observed for both mock and challenged fish, compared to controls (**Supplementary Table 2**). Regarding thrombocytes, a decrease was recorded at 6 h in infected fish compared to control and mock-challenged fish (**Supplementary Table 2**).

### Innate humoral parameters

An increase in plasma antiprotease was only observe in infected fish at 48 and 72 h post-challenge (**Supplementary Table 3**). For plasma protease activity, a peak was reached at 24 h followed by a decrease at 48 h post-challenge for infected sea bass, returning to values similar to those from controls after 72 h (**Supplementary Table 3**). Although not significant, a similar pattern was observed for mock-challenge fish at 24 h post-challenge (**Supplementary Table 3**). Plasma peroxidase activity also increased over time reaching a peak at 48 h post-challenged for both mock-challenged and infected fish (**Supplementary Table 3**), with both groups showing similar patterns of activity. A strong decrease in lysozyme activity was observed in infected fish from 24-72 h post-challenge, with a minimum at 48 h (**Supplementary Table 3**). A similar tendency to decrease was seen for mock-challenged fish, although the values in infected fish at 24, 48 and 72 h were much lower than in mock-challenged animals. Plasma bactericidal activity decreased in infected sea bass from 6 to 48 h post-challenge, with values lower than the ones obtained for mock-challenged fish (**Supplementary Table 3**). For plasma NO levels, no significant differences were found between mock and challenged groups, despite an increase was observed at 48 h post-challenge for both groups when compared with control values (**Supplementary Table 3**).

### Oxidative stress biomarkers

Hepatic catalase activity decreased over time until the end of the trial for both mock-challenged and challenged fish, with the two groups presenting similar values for each time point (**Supplementary Table 4**). Superoxide dismutase activity in liver was significantly higher for infected sea bass compared to control and mock-challenged fish at 6 h returning to basal values at the end of the trial (**Supplementary Table 4**). For mock-challenged specimens, a peak of activity was reached at 48 h post-challenge (**Supplementary Table 4**). No differences between time points were observed regarding lipid peroxidation, however, values were significantly lower for infected fish at 6 and 48 h post-challenge compared to mock-challenged group (**Supplementary Table 4**). Hepatic glutathione-S-transferase levels decreased slightly in challenged fish until the end of the trial, reaching its minimum value at 72 h post-challenge (**Supplementary Table 4**). Although a general tendency to decrease was also seen for mock-challenged fish, the values the values did not differ much from the basal ones (**Supplementary Table 4**).

Hepatic reduced glutathione decreased significantly between the control fish and both mock-challenged and infected fish after 72 h (**Supplementary Table 4**). Oxidized glutathione decreased until 48 h post-challenged in the liver of mock-challenged fish, and returned to basal values at 72 h. No differences were recorded for challenged fish (**Supplementary Table 4**). Regarding reduced:oxidized glutathione ratio in liver, no differences were recorded for challenged fish, but it was possible to distinguish a wave-like variation with a peak at 48 h post-challenge. The same was seen in the mock-challenge group, with a significantly higher value at that sampling time point, when compared to controls (0h) (**Supplementary Table 4**).

### Histology and immunohistochemistry analyses

The fish sampled before the trial for screening purposes did not display any histopathological changes and the same was recorded for the individuals from the control and mock-challenge groups.

**Figure 2:**
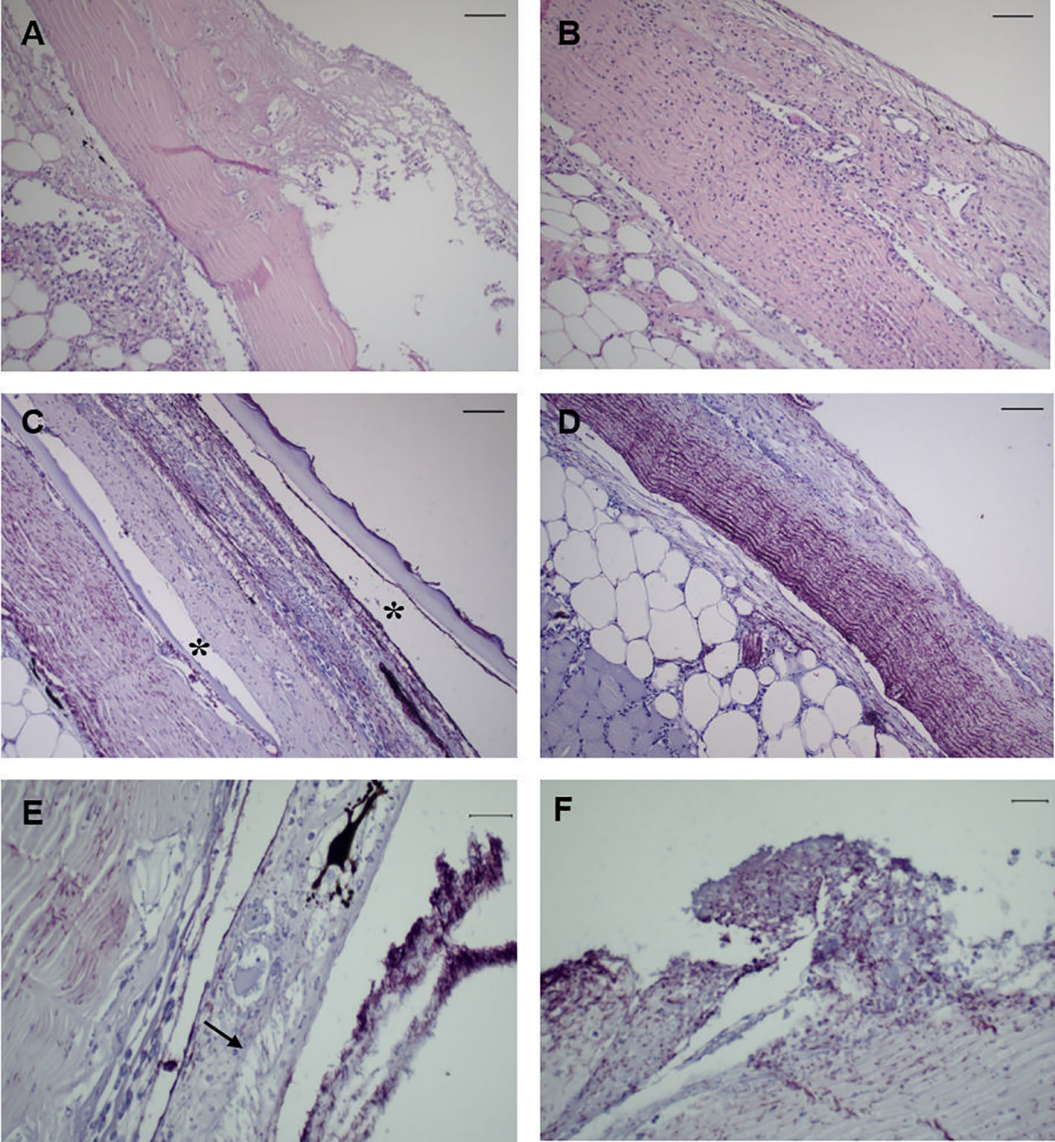
Representative images of skin tissue from European sea bass (*Dicentrarchus labrax*) bath-challenged with 5 x 10^5^ CFU mL^-1^ *T. maritimum*. **(A)** Heavy infiltration of inflammatory cells in the dermis of challenge fish at 24 h. H–E. Bar 50 μm. **(B)** Extensive necrosis of the dermis associated with infiltration of inflammatory cells in the hypodermis of challenge fish at 24 h. H–E. Bar 50 μm. **(C)** Immunohistochemistry against *T. maritimum* antigen, revealing extensive proliferation of *T. maritimum* in the dermis of challenged fish at 24 h, with agglomerates of bacteria in the epidermis and scale pockets (*) Bar 50 μm. **(D)** Necrosis and agglomerates of bacteria in the dermis with infiltration of inflammatory cells in the hypodermis. Bar 50 μm. **(E)** Vacuolization of epithelial cells from the epidermis of challegend fish at 24 h (arrrow), with agglomerates of *T. maritimum* in the same area. Bar 50 μm. **(F)** Proliferation of these bacteria in challenged fish at 48 h post-challenge. Bar 20 μm. Section subjected to immunocytochemistry against *T. maritimum* antigen.

No histopathological changes were observed in the analyzed mucosal organs at 6 h post-challenge for bacteria-challenged fish. Instead, infected fish started to display typical tenacibaculosis symptoms at 24 h post-challenge, with ulcers in different areas of the skin and frayed fins. At 24 h the lesions in the skin of bath-challenged fish showed similar degrees of severity, presenting a considerable number of scattered inflammatory cells in the dermis and hypodermis, with severe necrosis of the dermis and detachment or loss of the epidermis (**Figures 2A, B**). In the samples of the remaining organs, no evidence of histopathological changes was observed for any of the specimens analyzed through sampling time points. Immunoreactivity was detected only in the skin of infected fish at 24 h and 48 h post-challenge, being mainly distributed across the dermis, revealing an extensive and fast progression of the bacteria; along with necrosis and vacuolization, it was possible to observe the recruitment of inflammatory cells to adjacent areas at 24 h (**Figures 2C–F**). No immunoreactivity was detected in the remaining organs for any specimens through sampling time points.

### Gene expression analyses

#### Gills

**Figure 3:**
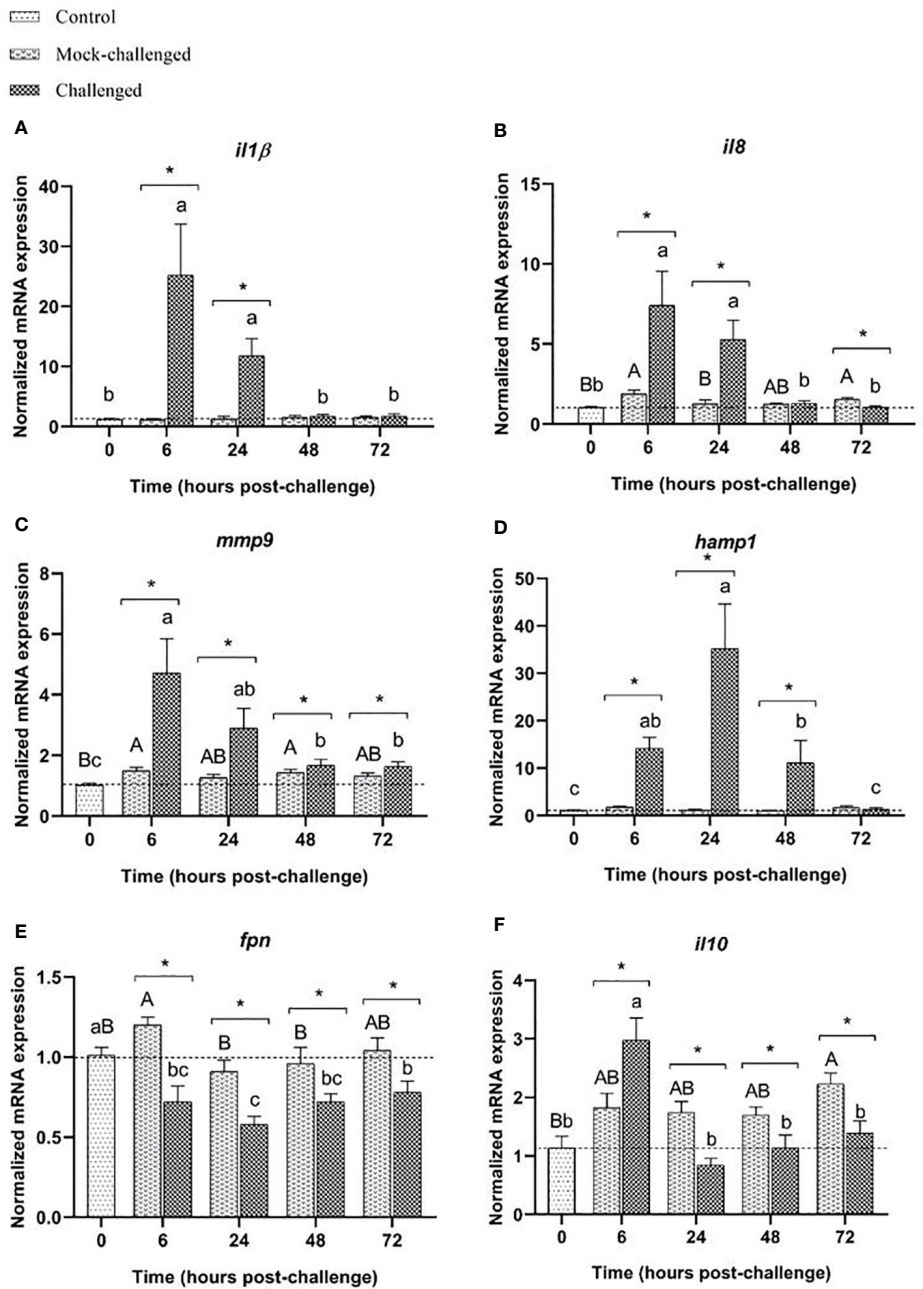
Expression of **(A)** *il-1β*, **(B)** *il8*, **(C)** *mmp9*, **(D)** *hamp1*, **(E)** *fpn* and **(F)** *il10* in gills of European sea bass (*Dicentrarchus labrax*) after bacterial bath-challenge with 5 x 10^5^ CFU mL^-1^ *T. maritimum*. Data are expressed as mean ± SEM (n=12 per treatment). Different capital letters indicate differences between control and mock-challenge and lower case letters indicate significant differences between control and challenged groups, while (*) represents statistical differences between mock and challenged fish at each sampling point (One-way ANOVA or Kruskal-Wallis; p ≤ 0.05).

Infected sea bass displayed a greater than 23-fold increase in the expression of the pro-inflammatory cytokine *il-1β* in the gills at 6 h post-challenge. At 24 h the expression was 9-fold higher than the expression in mock-challenged fish and returned to basal values after that sampling point (**Figure 3A**). A very similar pattern was also seen for *il8* and *mmp9* transcripts (**Figures 3B, C**). A high increase of *hamp1* expression was observed at 6, 24 and 48 h post-challenge in infected sea bass compared to control and mock-challenged fish, with a 30-fold peak at 24 h (**Figure 3D**). On the contrary, a slight, albeit significant decrease in *fpn* expression was noticed in infected specimens at all sampling points compared to control and mock-challenged fish (**Figure 3E**). The expression of the anti-inflammatory cytokine *il-10* did not change in the mock-challenged fish, but was increased at 6 h post-challenge for the infected ones (**Figure 3F**).

The expression of the *tlr2*, *tlr9*, *nod1* and *nod2* receptors in the gills did not change significantly after bacterial exposure when compared to controls (0 h), although a tendency to decreased *tlr2* expression was seen at 6 and 24 h post-infection (**Supplementary Table 5**). The mock-challenged fish showed higher *tlr2* expression than the control or challenged fish at all-time points (**Supplementary Table 5**). Regarding *tlr9* transcripts, the different treatment groups presented a similar pattern, with a downregulation at 24 h for infected fish (**Supplementary Table 5**). For the intracellular receptor *nod1*, an upregulation was observed at 6 h for challenged fish, returning afterwards to basal expression values (**Supplementary Table 5**). On the other hand, *nod2* suffered a significant downregulation in infected fish compared to control and mock-challenged groups, with the lower expression registered at 24 h post-challenge. Both transcription factors, *nf-κB* and *stat3*, presented higher expression values at 6 h post-challenge for infected fish followed by a decrease in the remaining time points (**Supplementary Table 5**). In the mock-challenged fish, no changes in the *nf-κB* expression were observed (**Supplementary Table 5**). The same pattern of expression was seen for *stat3* (**Supplementary Table 5**). Regarding *bcl2-like*, the expression in mock-challenged fish did not differ from the expression in control fish, but infected sea bass showed decreased expression throughout all sampling points (**Supplementary Table 5**). Expression of *il-6* was downregulated in infected fish at 48 and 72 h post-challenge compared to control and mock-challenged animals (**Supplementary Table 5**). The mock-challenged fish did not present any major differences for *il-6* expression. For the pro-inflammatory cytokine *tnfα*, the expression values for mock-challenged sea bass increased until the end of the time-course trial reaching its maximum at 72 h post-challenge, whereas a tendency to decrease was observed in the infected fish from 24 h onwards (**Supplementary Table 5**).

#### Skin

**Figure 4:**
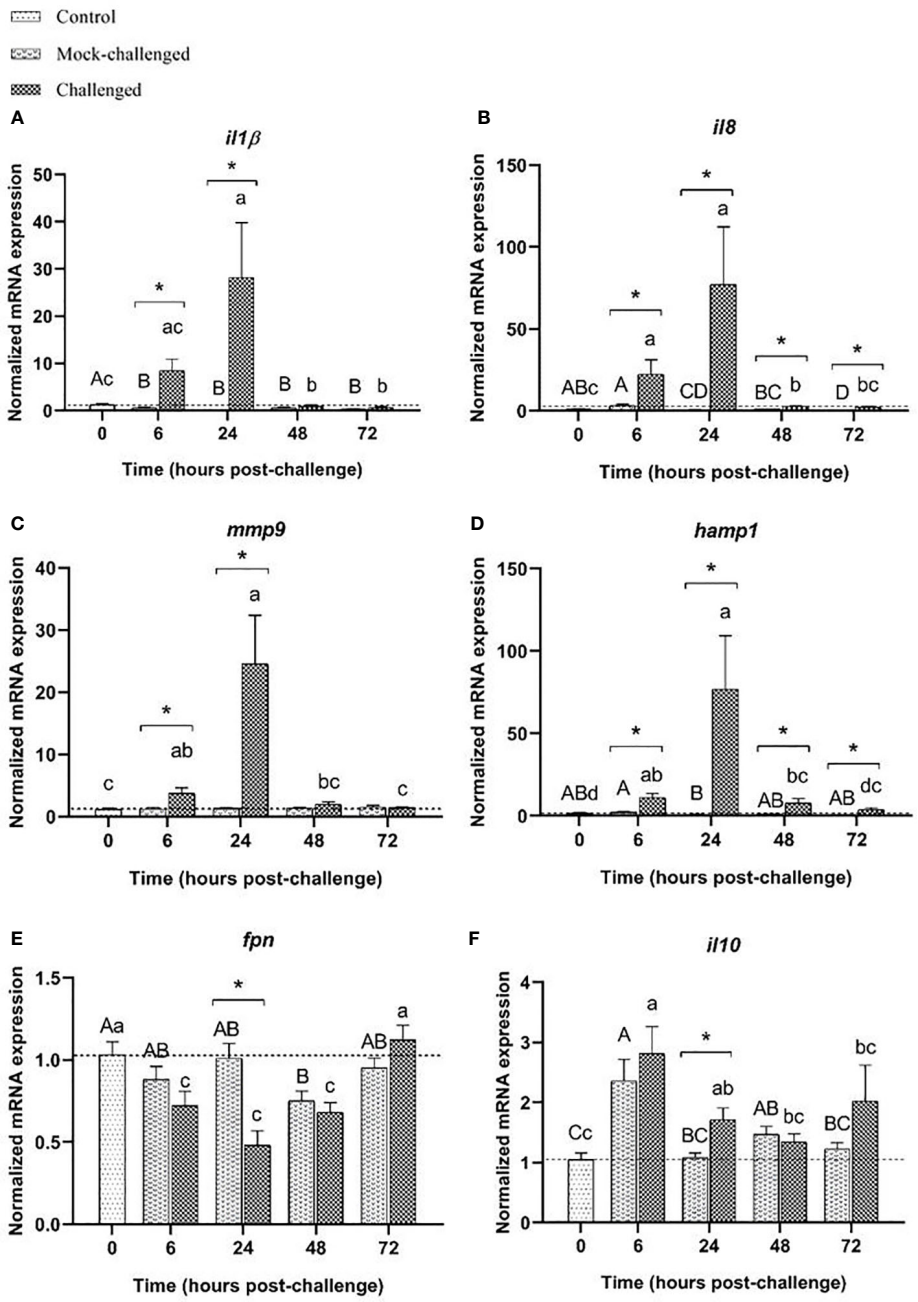
Expression of **(A)** *il-1β*, **(B)** *il8*, **(C)** *mmp9*, **(D)** *hamp1*, **(E)** *fpn* and **(F)** *il10* in the skin of European sea bass (*Dicentrarchus labrax*) after bath-challenge with 5 x 10^5^ CFU mL^-1^ *T. maritimum*. Data are expressed as mean ± SEM (n=12 per treatment). Different capital letters indicate differences between control and mock-challenge and lower case letters indicate significant differences between control and challenged groups, while (*) represents statistical differences between mock and challenged fish at each sampling point (One-way ANOVA or Kruskal-Wallis; p ≤ 0.05).

In what concerns the expression of pro-inflammatory mediators, the skin responded similarly to the gills. An upregulation of *il-1β* was observed at 6 h post-challenge in infected sea bass, with the higher expression (22-fold increase relative to control) recorded at 24 h (**Figure 4A**). A similar response was seen for *il8* and *mmp9* transcripts (**Figures 4B, C**). The antimicrobial peptide *hamp1* registered a significant increase in mRNA levels at 6, 24 and 48 h post-challenge for infected fish, reaching its maximum expression value at 24 h post-challenge with a 90-fold increase compared to mock-challenged fish (**Figure 4D**). Regarding *fpn* mRNA expression an opposite pattern was recorded, with a significant downregulation at 6, 24 and 48 h post-challenge for the infected group, returning to basal expression values after 72 h (**Figure 4E**). The expression of the anti-inflammatory cytokine *il-10* showed a significant, albeit moderate increase at 6 h post-challenge for both mock-challenged and infected groups, followed by a decrease at later time points to values similar to the basal level (**Figure 4F**).

As in the gills, no major differences in the expression of the studied cell receptors were observed in the skin of sea bass bath exposed to *T. maritimum*. Infected fish showed a decrease in *tlr2* transcripts at 6 and 24 h compared to the control group, and a higher expression at 72 h (**Supplementary Table 6**), whereas a tendency to increase was observed in the mock-challenged fish. No differences in the expression of the intracellular cell receptor *tlr9* were recorded (**Supplementary Table 6**). The *nod1* expression was slightly down-regulated in the mock-challenge group from 24 onwards. For the bath-challenged fish, *nod1* expression was decreased at 48 h and 72 h post-challenge (**Supplementary Table 6**). Regarding *nod2* expression levels, a wave pattern was observed in infected fish, with a significant decrease at 24 h followed by an upregulation at 48 and 72 h when compare to basal levels. In the case of mock-challenged fish, an upregulation of *nod2* was seen from 24 h onwards (**Supplementary Table 6**). The *nf-κB* mRNA levels were increased at 6 and 24 h infected fish, but decreased at 48 and 72 h to levels lower than the control ones. The mock-challenged fish had lower transcripts throughout the time-course study compared to the controls (**Supplementary Table 6**). The same mRNA expression pattern was observed for *stat3* (**Supplementary Table 6**). In the case of *bcl-2like*, a downregulation was seen throughout the time-course, especially at 24 h post-challenge for the infected fish compared to both control and mock-challenged groups. No differences were detected for mock-challenged ones (**Supplementary Table 6**). Mock-challenged and infected groups presented a similar pattern of *il-6* expression, with a significant upregulation at 6 h post-challenge, followed by a decrease in later time-points (**Supplementary Table 6**). No significant differences in *tnfα* were recorded in mock-challenged and infected fish, despite a tendency to increase in the infected group (**Supplementary Table 6**).

#### Posterior-intestine

**Figure 5:**
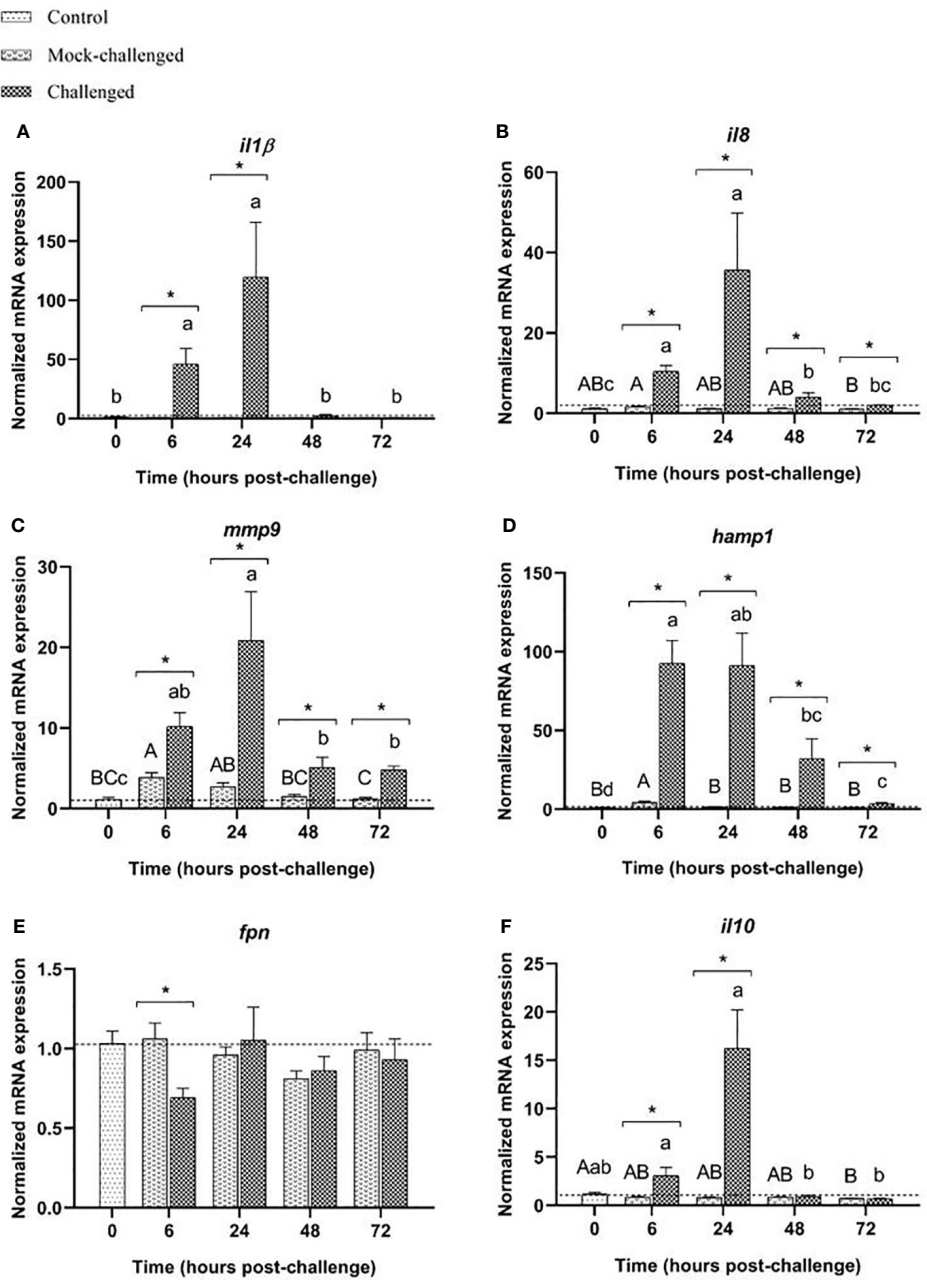
Expression of **(A)** *il-1β*, **(B)** *il8*, **(C)** *mmp9*, **(D)** *hamp1*, **(E)** *fpn* and **(F)** *il10* in posterior-intestine of European sea bass (*Dicentrarchus labrax*) after bacterial bath-challenge with 5 x 10^5^ CFU mL^-1^ *T. maritimum*. Data are expressed as mean ± SEM (n=12 per treatment). Different capital letters indicate differences between control and mock-challenge and lower case letters indicate significant differences between control and challenged groups, while (*) represents statistical differences between mock and challenged fish at each sampling point (One-way ANOVA or Kruskal-Wallis; p ≤ 0.05).

A clear increase in the levels of *il-1β* transcripts was recorded at 6 and 24 h in infected sea bass (46-fold and 126-fold increase, respectively compared to mock-challenged fish), similar to what was observed in the gills and skin (**Figure 5A**). The same patter occurred for *il8* (**Figure 5B**), and *mmp9* (**Figure 5C**), with an upregulation in infected sea bass at all-time points compared to control and mock-challenged groups. The antimicrobial peptide *hamp1* showed marked increase in expression in challenged fish at 6 h (20-fold increase relative to mock fish), slowly decreasing after this time point, despite infected sea bass presented much higher values than the control or mock-challenged groups at all-time points (**Figure 5D**). While *fpn* transcripts presented a tendency to be downregulated, especially for infected sea bass at 6 h post-challenge, no significant differences were found (**Figure 5E**). The cytokine *il-10* expression demonstrates a slightly different expression pattern to what was observed for the gills and skin response, with a significant increase at 24 h post-challenge (20-fold increase relative to mock-challenge). At 72 h post-challenge, values from infected fish returned to basal levels, similar to control ones (**Figure 5F**). Similarly, to what was seen in the gills and skin, no major differences in the expression of the studied cell receptors were observed in the posterior-intestine. Expression of *tlr2* presents was slightly decreased in the challenged fish at 6 and 24 h, returning to basal values a 72 h. No differences were seen for mock-challenged group (**Supplementary Table 7**). Regarding *tlr9* expression, no changes were detected in infected specimens when compared to controls, and a minor increase was recorded for mock-challenged fish at all time-points (**Supplementary Table 7**). For both *nod1* and *nod2*, no major differences in expression were noticed in infected animals. In mock-challenged fish, an increased *nod2* expression level was seen at 24 h post-challenge compared to control fish (**Supplementary Table 7**). Expression of *nf-κB* decreased from 24 h onwards in infected fish, and was also decreased in mock-challenged fish at all-time points (**Supplementary Table 7**). No differences in *stat3* expression were noticed in challenged and mock-challenged groups, when compared to controls (**Supplementary Table 7**). The expression of *bcl2-like* in mock-challenged and infected groups was slightly lower than in control fish at all-time points (**Supplementary Table 7**). For *il-6* mRNA levels no major differences were record (**Supplementary Table 7**). Regarding *tnfα*, no differences were recorded for mock-challenged fish, but a decreased expression was observed at 24 h post-challenge for infected fish, compared to control and mock-challenged groups (**Supplementary Table 7**).

## Discussion

With its ubiquitous distribution in marine environments, *T. maritimum* restrains the rearing of numerous fish species (46) and is considered one of the most threating bacterial infections (47) for aquaculture. However, available knowledge regarding *T. maritimum* pathogenesis is very limited. The present study evaluated cellular, humoral, oxidative and molecular short-term responses of European sea bass following *T. maritimum* bath challenge, providing an insight of the host’s responses against this particular bacterium.

In 1990s a few pathogenicity studies were developed with commercial fish species, which included European sea bass (48), Atlantic salmon (49), rainbow trout, and greenback flounder (*Rhombosolea tapirina*) (50), resulting in distinctive rates of cumulative mortality. Initially, prolonged immersion challenges of 18 h proved to be effective in reproducing tenacibaculosis in turbot (51). More recently, Mabrok et al. (25) also used a prolonged bath of 24 h at 23°C to successfully challenge Senegalese sole with different strains of *T. maritimum* (ACC6.1, ACC13.1 and ACC20.1), resulting in cumulative mortalities ranging from 50% to 100%. In this same study, the ACC13.1 *T. maritimum* strain (the same used in the present study) (9.6 × 10^5^ cells mL^−1^) lead to cumulative mortalities of approximately 50% in Senegalese sole. In the last years, immersion challenge has been frequently used as a reliable method to experimentally reproduce tenacibaculosis in fish (52–57). In the present study, a mortality rate of 32.1% was obtained for the bath-challenged fish, while displaying tenacibaculosis clinical signs (e.g. ulcerative lesions in the skin and caudal fins, with haemorrhages, loose scales and abrasions). Since this challenge model was also able to successfully induce tenacibaculosis clinical signs and mortality in the bacteria exposed fish, it is suggested that immersion challenge (with a 2 h period of bacterial exposure) is an effective method to experimentally reproduce this disease in European sea bass. Similar mortality traits were also observed in previous studies following the same immersion challenge procedure (56, 57).

Fish bath-challenged with *T. maritimum* presented a moderate decrease in total RBCs counts and a decreased haematocrit, suggesting the occurrence of anemia in response to infection. Further studies are required to elucidate if the observed anemia results from bacterial-induced destruction of RBCs or to an insufficient supply of healthy RBCs. The complete genome sequence of *T. maritimum* was able to offer some insights about virulence/associated genes which encode the biosynthesis of hemolysins (19). The secretion of these hemolysins may be a possible explanation for the decrease of both RBCs and haematocrit in fish exposed to *T. maritimum*. However, these haematological parameters can also suggest anemia of inflammation, a host’s off-target strategy, where erythrocyte lifespan is shortened by activating macrophages allowing the sequestration of iron from serum by these cells (58, 59).

Analysis of the MCH showed that it increased after bacterial challenge, revealing a higher amount of hemoglobin inside the RBCs, also supported by a slight increase in MCHC. As previously mentioned, the presence of *T. maritimum* hemolysins may lead to RBCs lysis, which can lead to increased RBC production secondary to peripheral blood cell destruction, with the formed cells carrying more hemoglobin than normal-sized cells (60). Moreover, these incompletely processed RBCs, are slightly larger than the average RBC, increasing theses red cell indices (61).

Neutrophils are responsible to assemble an early and potent antimicrobial response against invading pathogens, being the first leukocytes to be recruited to inflammatory sites (62, 63). Even though blood total leucocyte numbers did not change in response to bath-challenge with *T. maritimum*, there was an increase in neutrophils at 48 and 72 h, suggesting that they are involved in the response to *T. maritimum* infection. These data are in agreement with the observed increased expression of the pro-inflammatory cytokines at the mucosal organs already at 6 h following infection. IL-8 has a potent chemotropic activity for neutrophils, monocytes, basophils and other immune cells (64), and several studies have demonstrated that increased expression of *il-8* is related to acute inflammatory responses in teleosts upon infection with different bacterial species (65–68). Therefore, it is likely that the upregulation of *il-8* expression in the mucosal organs of infected fish is related with the infiltration of inflammatory cells in the skin lesions, detected at 24 h post-challenge. The results obtained in the current study are in agreement with previous results obtained by Guardiola et al. (22) in Senegalese sole, which revealed a significant increase of neutrophils at 48 and 72 h after bath-challenge with *T. maritimum* (22), as well as with results reported in other studies for several bacterial fish pathogens (69–71).

Moreover, besides being a key mediator of the immune system, neutrophils are also the main responsible for the production of myeloperoxidase in the plasma (72). Therefore, the increase of peroxidase in the plasma of infected specimens from the present study can be mostly explained by the neutrophilia observed in bath-challenged fish.

During inflammation, circulating monocytes migrate to infection sites, following conditioning by pro-inflammatory cytokines, microbial products and local growth factors, differentiating afterwards into macrophages and dendritic cells (73). The increased number of monocytes at 72 h post-challenge may indicate a host’s attempt to increase the number of monocytes and their macrophage and dendritic-cell progeny, to fight against invading pathogens through phagocytosis, assist in the repair/regenerate of the damaged tissue, resolve the inflammation as well as to stablish the link with adaptive immunity by antigen presentation (74–76).

Studies have demonstrated that thrombocytes are involved in innate immune and inflammatory responses in fish, participating in phagocytic activities and in the killing of internalized bacteria (77–79). In the present study, the successive increase in circulating thrombocytes in bath-challenged fish up to 24 h following infection, allows to hypothesized that these cells are also migrating to infection sites. Since the teleost adaptive immune system implicates slow proliferation and maturation of lymphocytes (80, 81), their role in this time frame may not be as relevant as the other immune cells, as seen by the lack of variation during the first 72 h post-challenge.

Plasma lysozyme activity decreased in infected fish, whereas bactericidal activity in the plasma only started to increase upon 48 h following bacterial bath challenge, which can be related with the late influx of phagocytes (e.g. neutrophils and monocytes) (82) at the end of the time-course study. Many Gram-negative bacteria are able to produce lysozyme inhibitors that can significantly inhibited/decrease lysozyme activity in the host serum beginning from the early stages of host infection (83). Therefore, more studies would be needed to understand if *T. maritimum* could have similar evading mechanisms.

Pathogenic bacteria must be able to adapt to unpredictable environments and to cope with diverse stress-inducing factors, such as reactive oxygen species (ROS) produced by the host’s macrophages (19). *T. maritimum*’s genome encodes three different superoxide dismutases (SodA, SodB, and SodC) and two catalases/peroxidase (KatA and KatG), which may imply that these bacteria use a complex mechanism to fight oxidative stress (19). Therefore, it is tempting to speculate that *T. maritimum* is able to use these enzymes to cope with and to modulate the host’s immune response, resulting in the lack of changes regarding the analyzed oxidative stress parameters.

In order to recognize bacteria, the host’s immune system rely on pattern recognition receptors (PRRs), which are able to bind and recognize different pathogen-associated molecular patterns (PAMPs) and activate immune cells (84, 85).

Although no major changes in the expression of the studied PRRs and transcription factors in response to *T. maritimum* infection were detected in the present study, an immune response was indeed developed in bath-challenged fish, as a clear pro-inflammatory response was observed across all mucosal organs analyzed.

Several studies revealed that, when challenged with pathogenic bacteria, teleosts upregulate the expression of *il-1β* as initiation of the non-specific inflammatory response (66, 86, 87), with similar expression kinetics to the present study. The same upregulation is seen for *mmp9*, which increased expression was already linked to immune response against *Listeria monocytogenes* in infected zebrafish (88), *Flavobacterium psychrophilum* in rainbow trout (*Oncorhynchus mykiss*) (89), *Aeromonas hydrophila* in yellow catfish (*Pelteobagrus fulvidraco*) (90), and in peritoneal and peripheral blood leucocytes of stimulated common carp (*Cyprinus carpio*) (91). The skin and posterior-intestine of challenged fish responded quite similarly, with *il-1β*, *il8* and *mmp9* as the most highly induced genes, which provides evidence that an inflammatory response is activated upon infection with *T. maritimum*. The increase in *il-1β* expression in these mucosal organs may result in increased mucus secretion (92, 93), which could be advantageous during a *T. maritimum*’s infection. The increased expression of *il8* and *mmp9* can also be intertwined with *il-1β* expression, since this cytokine is able to promote the release of other cytokines and activate macrophages and other immune cells (82, 94). Not only *T. maritimum* was able to trigger a pro-inflammatory response in the host, but also modulated the expression of genes related to iron metabolism regulation. The response of mucosal organs may suggest that one of the mechanisms employed by the host to withstand *T. maritimum* is associated with hepcidin, a small antimicrobial peptide that is involved in iron metabolism regulation in mammals (95–97). The iron control in the extracellular environment is a known innate immune strategy developed to deprive pathogens of iron, an essential nutrient for bacterial growth, replication, and metabolic processes (98). This strategy, as a response to inflammatory stimuli, leads to high circulating levels of hepcidin, which in turn, negatively regulate the iron concentration in plasma (99) through occlusion of the open-outward conformation (100) or by internalization and degradation of ferroportin, an iron exporter (101, 102). Although there was no clear activation of the pro-inflammatory cytokine IL-6, the main hepcidin inducer (103), other experiments already revealed that hepcidin can also be induced by IL-1β (104, 105). Therefore, it is possible that in this study, hepcidin regulation was due to the inflammatory signals conducted by IL-1β, with the activation of NF-κB and JNK signaling pathways (105), leading to the seen transcriptional *hamp* induction and to the downregulation of *fpn*. The previously described mild anaemic condition of challenged fish, that can be referred to as anemia of inflammation, could have some repercussions in the expression levels of hepcidin, since in an anaemic situation, hepcidin synthesis is suppressed (97, 106, 107). However, this type of response was also recorded by 108, where *Photobacterium damselae* spp. *piscicida* challenged fish demonstrated that hepcidin responds to infection by increasing its expression levels in sea bass liver, despite the anaemia demonstrated by the infected fish. This hepcidin dynamic was already described for other fish species (109–112). These results are in accordance with other studies that also demonstrated the regulation of hepcidin and ferroportin by inflammatory signals induced by a pathogen (108, 113–116).

Although it is known that IL-1β is typically activated in situations where TNF-α is produced, no changes were detected in its expression after challenging sea bass with *T. maritimum* (117, 118). In a study developed by Nascimento et al. (119), a TNF-α up-regulation was briefly observed at 12 h post-infection with *Photobacterium damselae* subsp. *piscicida* in the head-kidney of European sea bass (119). Another study with *Streptococcus iniae*, presented the same results as the previous one for the head-kidney of European sea bass (120). Also, *tnfα* was significantly increased from 6 to 9 h post-infection in the head-kidney of European sea bass when intramuscularly infected with Betanodavirus (RGNNV), showing an early response of this gene (121).

Since *tnf-α* and *il-6* did not show any major differences in their expression, an earlier sampling time point could be valuable to add information about their possible upregulation in a response against *T. maritimum*.

Due to its ubiquitous distribution and lack of host specificity, *T. maritimum* continuously inflicts significant losses among cultured marine species, as confirmed by the re-emerging nature of tenacibaculosis outbreaks in salmon farms globally (122). Although some progress has been made in the last decade regarding its pathogenic mechanisms, defining the host-pathogen relationship has proved to be very difficult to achieve. The present study offers a new insight regarding the mucosal innate immune response upon a pathogen inoculation pathway that mimics natural infection dynamics. In summary, the kinetics of the expression of molecular immune markers in gill, skin and posterior intestine of bath-challenged fish together with the findings observed for peripheral leucocytes demonstrate the occurrence of a pro-inflammatory response against *T. maritimum* in the studied mucosal organs, with a faster kinetic in the gills, which may suggest that this pathogen can use gill mucosa as a route of entry into the fish. The analysis of the humoral parameters suggests that the local response at the mucosal organs is followed by a response at systemic level.

## Data availability statement

The data presented in the study are deposited in the “Figshare” repository, DOI 10.6084/m9.figshare.23998017.

## Ethics statement

The animal study was reviewed and approved by 0421/000/000/2020. The study was conducted in accordance with the local legislation and institutional requirements.

## Author contributions

IF: Formal Analysis, Methodology, Writing – original draft. DP: Methodology, Writing – review & editing. AL: Methodology, Writing – review & editing. MQ: Supervision, Writing – review & editing. AV: Conceptualization, Supervision, Writing – review & editing. BC: Conceptualization, Supervision, Writing – review & editing.
