# Supplementary material for: Early innate immune responses in European sea bass (Dicentrarchus labrax L.) following Tenacibaculum maritimum infection
Source: Front Immunol. 2023 Sep 4;14:1254677. doi: 10.3389/fimmu.2023.1254677 (PMC10507263; doi:10.3389/fimmu.2023.1254677)
Supplement: Supplementary file 1 [file DataSheet_1.docx]

***Supplementary materials***

**Table 1:** Haematological parameters **(**red blood cells (RBC x 10^6^/µL), white blood cells (WBC x 10^4^/µL), haematocrit (Ht %), haemoglobin (Hg, g/dL), mean corpuscular volume (MCV µm^3^), mean corpuscular haemoglobin (MCH, pg/cell) and mean corpuscular haemoglobin concentration (MCHC, g/100 mL)) of European sea bass (*Dicentrarchus labrax*) after bacterial bath-challenge with 5 x 10^5^ CFU mL^-1^ *T. maritimum*. Data are expressed as mean ± SEM (n=12 per treatment). Different capital letters in the same row stand for differences between control and mock-challenge and lower case letters indicate significant differences between control and challenged groups, while (*) represents statistical differences between mock and challenged fish at each sampling point (One-way ANOVA or Kruskal-Wallis; p≤0.05).

|  | **Control** | **Mock-challenged** | | | | **Challenged** | | | |
| --- | --- | --- | --- | --- | --- | --- | --- | --- | --- |
|  | **0 h** | **6 h** | **24 h** | **48 h** | **72 h** | **6 h** | **24 h** | **48 h** | **72 h** |
| **RBC**  **(x10^6^/µL)** | 2.47± 0.06^Aa^ | 2.53±0.07^A*^ | 2.49±0.10^A*^ | 2.03±0.14^B*^ | 2.60±0.13^A^ | 1.85±0.07^b*^ | 2.10±0.08^b*^ | 1.58±0.04^c*^ | 2.34±0.07^a^ |
| **WBC (x10^4^/µL)** | 2.48±0.22^Bb^ | 2.72±0.34^AB^ | 2.93±0.20^AB*^ | 2.70±0.16^AB^ | 3.66±0.32^A^ | 2.05±0.26^b^ | 2.23±0.20^b*^ | 2.47±0.22^b^ | 4.20±0.31^a^ |
| **Ht (%)** | 32.17±0.64^Bab^ | 35.58±0.94^B*^ | 36.75±1.73^AB*^ | 35.90±1.68^AB*^ | 41.27±1.28^A*^ | 30.58±1.21^ab*^ | 29.50±1.63^ab*^ | 28.92±1.00^b*^ | 33.36±0.75^a*^ |
| **Hg (g/dL)** | 1.44±0.05^B^ | 1.61±0.07^AB*^ | 1.56±0.06^AB^ | 1.37±0.09^B^ | 1.84±0.09^A^ | 1.37±0.06^*^ | 1.66±0.07 | 1.46±0.08 | 1.55±0.12 |
| **MCV (µm^3^)** | 130.63±2.73^c^ | 141.54±4.39^*^ | 148.48±5.85 | 169.23±12.58 | 161.63±11.05 | 167.37±9.11^ab*^ | 142.22±8.99^abc^ | 168.56±3.97^a^ | 147.51±5.35^bc^ |
| **MCH (pg/cell)** | 5.87±0.26^Bc^ | 6.38±0.25^AB*^ | 6.33±0.26^AB*^ | 6.31±0.36^AB*^ | 7.29±0.36^A^ | 7.40±0.28^b*^ | 7.94±0.35^ab*^ | 9.15±2.76^a*^ | 6.61±0.45^bc^ |
| **MCHC**  **(g/100 mL)** | 4.50±0.20^b^ | 4.54±0.19 | 4.32±0.22^*^ | 4.06±0.25^*^ | 4.61±0.39 | 4.50±0.20^b^ | 5.70±0.26^a*^ | 5.14±0.36^ab*^ | 4.57±0.39^ab^ |

**Table 2:** Absolute values (x 10^4^/µL) of peripheral blood leukocytes (neutrophils, monocytes, lymphocytes and thrombocytes) of European sea bass (*Dicentrarchus labrax*) after bacterial bath-challenge with 5 x 10^5^ CFU mL^-1^ *T. maritimum*. Data are expressed as mean ± SEM (n=12 per treatment). Different capital letters in the same row stand for differences between control and mock-challenge and lower case letters indicate significant differences between control and challenged groups, while (*) represents statistical differences between mock and challenged fish at each sampling point (One-way ANOVA or Kruskal-Wallis; p≤0.05).

|  | **Control** | **Mock-challenged** | | | | **Challenged** | | | |
| --- | --- | --- | --- | --- | --- | --- | --- | --- | --- |
|  | **0 h** | **6 h** | **24 h** | **48 h** | **72 h** | **6 h** | **24 h** | **48 h** | **72 h** |
| **Neutrophils (x10^4^/µL)** | 3.24E-02±0.01^Cc^ | 1.80E-01±0.03^A^ | 1.41E-01±0.03^AB*^ | 6.48E-02±0.02^BC*^ | 5.38E-02±0.01^BC*^ | 2.17E-01±0.03^b^ | 4.90E-02±0.01^c*^ | 1.53E-01±0.03^b*^ | 5.66E-01±0.06^a*^ |
| **Monocytes (x10^4^/µL)** | 8.42E-03±0.00^b^ | 1.51E-02±0.00 | 2.18E-02±0.01 | 9.55E-03±0.00^*^ | 1.58E-02±0.01^*^ | 2.05E-02±0.01^b^ | 1.91E-02±0.01^b^ | 6.73E-02±0.02^a*^ | 7.33E-02±0.02^a*^ |
| **Lymphocytes (x10^4^/µL)** | 1.05E+00±0.12^Bb^ | 1.33E+00±0.13^AB^ | 1.70E+00±0.13^A*^ | 1.04E+00±0.14^B^ | 1.83E+00±0.25^A^ | 1.39E+00±0.17^ab^ | 1.26E+00±0.14^b*^ | 8.64E-01±0.10^b^ | 1.83E+00±0.25^a^ |
| **Thrombocytes (x10^4^/µL)** | 1.37E+00±0.17^a^ | 1.19E+00±0.23^*^ | 1.07E+00±0.12 | 1.58E+00±0.16 | 1.44E+00±0.23 | 5.14E-01±0.10^b*^ | 9.05E-01±0.10^ab^ | 1.38E+00±0.19^a^ | 1.23E+00±0.15^a^ |

**Table 3:** Immune parameters (antiprotease (%) and proteases activities (%), peroxidase (units/mL), lysozyme (units/mL), bactericidal activity (%) and nitrite concentration (µM)) of plasma of European sea bass (*Dicentrarchus labrax*) after bacterial bath-challenge with 5 x 10^5^ CFU mL^-1^ *T. maritimum*. Data are expressed as mean ± SEM (n=12 per treatment). Different capital letters in the same row stand for differences between control and mock-challenge and lower case letters indicate significant differences between control and challenged groups, while (*) represents statistical differences between mock and challenged fish at each sampling point (One-way ANOVA or Kruskal-Wallis; p≤0.05).

|  | **Control** | **Mock-challenged** | | | | **Challenged** | | | |
| --- | --- | --- | --- | --- | --- | --- | --- | --- | --- |
|  | **0 h** | **6 h** | **24 h** | **48 h** | **72 h** | **6 h** | **24 h** | **48 h** | **72 h** |
| **Antiprotease (%)** | 96.31± 0.28^Bb^ | 97.38±0.47^AB^ | 97.77±0.28^A^ | 96.83±0.15^AB*^ | 96.91±0.30^AB*^ | 96.82±0.42^b^ | 90.87±4.22^b^ | 98.00±0.21^a*^ | 97.96±0.19^a*^ |
| **Protease (%)** | 10.46±0.69^ab^ | 10.12±0.67 | 11.28±0.73 | 9.07±0.46^*^ | 11.11±0.57^*^ | 8.69±0.27^bc^ | 12.52±0.90^a^ | 7.76±0.22^c*^ | 9.16±0.61^bc*^ |
| **Peroxidase (units/mL)** | 4.08±0.62^Bb^ | 2.58±0.31^B^ | 3.56±0.55^B^ | 9.93±1.28^A^ | 6.23±1.31^AB^ | 3.85±0.95^b^ | 5.27±0.95^ab^ | 8.80±1.34^a^ | 8.51±1.48^ab^ |
| **Lysozyme (units/mL)** | 7.64±0.87^ABa^ | 11.09±0.81^A*^ | 9.13±1.00^AB*^ | 8.10±1.19^AB*^ | 6.31±0.77^B*^ | 7.04±0.65^a*^ | 4.35±0.57^b*^ | 3.09±0.42^b*^ | 4.13±0.45^b*^ |
| **Bactericidal act (%)** | 26.13±2.49^Bab^ | 24.74±2.05^B*^ | 24.45±1.98^B*^ | 15.03±1.94^A^ | 31.82±2.65^AB*^ | 16.66±2.03^bc*^ | 9.80±2.09^c*^ | 14.36±1.68^c^ | 31.82±2.65^a*^ |
| **NO (µM)** | 0.11±0.01^Bc^ | 0.10±0.01^B^ | 0.13±0.01^B^ | 0.23±0.03^A^ | 0.18±0.02^A^ | 0.08±0.01^d^ | 0.13±0.02^bcd^ | 0.24±0.03^a^ | 0.17±0.01^ab^ |

**Table 4:** Oxidative stress biomarkers (catalase activity (CAT), superoxide dismutase activity (SOD), lipid peroxidation (LPO), glutathione-S-transferase (GST), reduced: oxidized glutathione ratio (GSH/GSSG ratio), reduced (GSH) and oxidized glutathione (GSSG) activity of liver of European sea bass (*Dicentrarchus labrax*) after bacterial bath-challenge with 5 x 10^5^ CFU mL^-1^ *T. maritimum*. Data are expressed as mean ± SEM (n=12 per treatment). Different capital letters in the same row stand for differences between control and mock-challenge and lower case letters indicate significant differences between control and challenged groups, while (*) represents statistical differences between mock and challenged fish at each sampling point (One-way ANOVA or Kruskal-Wallis; p≤0.05).

|  | **Control** | **Mock-challenged** | | | | **Challenged** | | | |
| --- | --- | --- | --- | --- | --- | --- | --- | --- | --- |
|  | **0 h** | **6 h** | **24 h** | **48 h** | **72 h** | **6 h** | **24 h** | **48 h** | **72 h** |
| **CAT (U/mg protein)** | 217.45±4.40^Aa^ | 173.54±14.75^AB^ | 156.38±5.79^B^ | 154.03±10.53^B^ | 137.37±6.44^B^ | 185.67±12.98^a^ | 139.30±9.93^b^ | 132.33±11.38^b^ | 139.55±9.70^b^ |
| **SOD (U/mg protein)** | 9.51±0.55B^c^ | 12.51±1.08^AB*^ | 13.41±0.87^A^ | 15.71±0.81^A*^ | 14.13±0.92A^*^ | 17.67±1.47a* | 14.64±4.54^ab^ | 12.57±0.71b^*^ | 11.64±0.51bc^*^ |
| **LPO (nmol/g wet tissue)** | 33.82±2.27 | 38.43±3.30^*^ | 36.53±4.12 | 50.11±5.67^*^ | 43.78±5.63 | 29.12±1.80^*^ | 33.36±1.91 | 28.98±2.56^*^ | 43.67±4.71 |
| **GST (nmol/mg protein)** | 194.90±8.93^ABa^ | 196.29±10.01^AB^ | 212.64±8.66A^*^ | 169.51±9.51^B^ | 162.14±12.09^B^ | 180.17±9.57^a^ | 181.29±9.35^a*^ | 167.10±6.86^ab^ | 130.71±9.64^b^ |
| **GSH/GSSG ratio** | 45.29±5.38^B^ | 37.42±4.56^B^ | 44.10±4.73^B^ | 82.56±14.33^A^ | 40.15±3.42^B^ | 39.45±4.69 | 54.19±4.80 | 60.94±13.59 | 42.54±3.20 |
| **GSH (µM)** | 6039.70±214.87^Aa^ | 5038.26±285.31^AB^ | 5320.99±328.45^AB^ | 5146.05±365.65^AB^ | 4373.64±272.44^B^ | 5133.91±450.02^ab^ | 5230.67±439.80^ab^ | 6129.85±504.89^ab^ | 4642.90±196.25^b^ |
| **GSSG (µM)** | 145.71±17.17^A^ | 138.74±9.72^A^ | 114.06±12.05^A^ | 65.20±8.36B^*^ | 107.82±6.33^A^ | 106.84±15.22 | 89.20±7.58 | 89.20±23.01^*^ | 109.77±9.22 |

**Table 5:** Quantitative expression of *tlr2*, *tlr9*, *nod1*, *nod2*, *nf-κB*, *stat3*, *bcl2-like*, *il-6* and *tnfα* for gills of European sea bass (*Dicentrarchus labrax*) after bacterial bath-challenge with 5 x 10^5^ CFU mL^-1^ *T. maritimum*. Data are expressed as mean ± SEM (n=12 per treatment). Different capital letters in the same row stand for differences between control and mock-challenge and lower case letters indicate significant differences between control and challenged groups, while (*) represents statistical differences between mock and challenged fish at each sampling point (One-way ANOVA or Kruskal-Wallis; p≤0.05).

|  | **Control** | **Mock-challenged** | | | | **Challenged** | | | |
| --- | --- | --- | --- | --- | --- | --- | --- | --- | --- |
| **Genes** | **0 h** | **6 h** | **24 h** | **48 h** | **72 h** | **6 h** | **24 h** | **48 h** | **72 h** |
| ***tlr2*** | 1.04±0.09^Bbc^ | 1.74±0.17^A*^ | 1.71±0.10^A*^ | 1.86±0.19^A*^ | 2.05±0.09^A*^ | 1.00±0.14^bc*^ | 0.64±0.14^c*^ | 1.28±0.12^ab*^ | 2.05±0.09^a*^ |
| ***tlr9*** | 1.01±0.05^Ba^ | 1.59±0.13^A*^ | 1.07±0.06^B*^ | 1.12±0.05^B*^ | 1.37±0.08^A*^ | 0.83±0.09^ab*^ | 0.61±0.07^c*^ | 0.68±0.04^bc*^ | 0.81±0.08^ab*^ |
| ***nod1*** | 1.02±0.06^Bb^ | 1.11±0.07^AB*^ | 0.99±0.09^B^ | 1.13±0.06^AB*^ | 1.40±0.09^A*^ | 1.57±0.13^a*^ | 1.03±0.09^b^ | 0.88±0.07^b*^ | 0.86±0.06^b*^ |
| ***nod2*** | 1.03±0.08^a^ | 0.95±0.04^*^ | 1.00±0.07^*^ | 1.00±0.04^*^ | 1.11±0.08^*^ | 0.72±0.11^bc*^ | 0.61±0.05^c*^ | 0.71±0.02^bc*^ | 0.82±0.06^ab*^ |
| ***nf-κB*** | 1.01±0.04^ABa^ | 1.01±0.05^AB*^ | 0.88±0.06^B^ | 0.99±0.05^AB*^ | 1.11±0.06^A*^ | 1.45±0.16^a*^ | 0.74±0.05^b^ | 0.68±0.03^b*^ | 0.78±0.05^b*^ |
| ***stat3*** | 1.02±0.06^Ab^ | 0.99±0.05^AB*^ | 0.80±0.06^B*^ | 1.01±0.06^A^ | 1.04±0.05^A^ | 2.22±0.19^a*^ | 1.25±0.06^b*^ | 1.08±0.08^b^ | 1.15±0.08^b^ |
| ***bcl2-like*** | 1.01±0.05^ABa^ | 0.98±0.04^AB^ | 0.87±0.06^B^ | 0.90±0.03^B*^ | 1.12±0.06^A*^ | 0.87±0.06^ab^ | 0.77±0.06^b^ | 0.69±0.03^b*^ | 0.69±0.04^b*^ |
| ***il-6*** | 1.03±0.08^ABa^ | 1.17±0.08^A^ | 0.89±0.08^B^ | 0.86±0.03^B*^ | 0.95±0.06^AB*^ | 1.15±0.10^a^ | 1.12±0.17^ab^ | 0.62±0.03^c*^ | 0.62±0.03^c*^ |
| ***tnfα*** | 1.04±0.09^Bab^ | 1.43±0.11^B*^ | 1.28±0.13^B*^ | 1.85±0.16^A*^ | 2.28±0.19^A*^ | 1.71±0.27^a*^ | 0.86±0.18^b*^ | 1.02±0.11^ab*^ | 0.82±0.09^b*^ |

**Table 6:** Quantitative expression of *tlr2*, *tlr9*, *nod1*, *nod2*, *nf-κB*, *stat3*, *bcl2-like*, *il-6* and *tnfα* for skin of European sea bass (*Dicentrarchus labrax*) after bacterial bath-challenge with 5 x 10^5^ CFU mL^-1^ *T. maritimum*. Data are expressed as mean ± SEM (n=12 per treatment). Different capital letters in the same row stand for differences between control and mock-challenge and lower case letters indicate significant differences between control and challenged groups, while (*) represents statistical differences between mock and challenged fish at each sampling point (One-way ANOVA or Kruskal-Wallis; p≤0.05).

| **Genes** | **Control** | **Mock-challenged** | | | | **Challenged** | | | | |
| --- | --- | --- | --- | --- | --- | --- | --- | --- | --- | --- |
|  | **0 h** | **6 h** | **24 h** | **48 h** | **72 h** | **6 h** | **24 h** | **48 h** | **72 h** |  |
| ***tlr2*** | 1.10±0.14^Bb^ | 1.09± 0.13^B^ | 1.62± 0.12^A*^ | 1.75± 0.14^A^ | 1.75± 0.18^A^ | 0.84± 0.13^c^ | 0.46± 0.12^c*^ | 1.56± 0.24^ab^ | 2.27± 0.23^a^ |  |
| ***tlr9*** | 1.05±0.10 | 1.20± 0.09^*^ | 1.11± 0.15^*^ | 0.82± 0.08 | 0.98± 0.08 | 0.89± 0.08^*^ | 0.72± 0.08^*^ | 0.81± 0.13 | 1.06± 0.13 |  |
| ***nod1*** | 1.10±0.15^Aab^ | 0.93± 0.10^AB^ | 0.70± 0.07^ABC*^ | 0.59± 0.06^C^ | 0.63± 0.06^BC^ | 1.27± 0.18^a^ | 1.20± 0.16^a*^ | 0.71± 0.07^bc^ | 0.68± 0.06^c^ |  |
| ***nod2*** | 1.13±0.20^Bb^ | 1.26± 0.15^B*^ | 2.43± 0.21^A*^ | 2.00± 0.17^A^ | 2.11± 0.21^A^ | 2.10± 0.22^a*^ | 1.16± 0.24^bc*^ | 1.64± 0.12^ac^ | 2.10± 0.31^a^ |  |
| ***nf-κB*** | 1.01±0.05^Ab^ | 0.78± 0.05^B*^ | 0.62± 0.04^B*^ | 0.70± 0.04^B^ | 0.70± 0.04^B^ | 1.29± 0.08^ab*^ | 1.38± 0.18^ab*^ | 0.75± 0.03^d^ | 0.90± 0.11^cd^ |  |
| ***stat3*** | 1.06±0.10^Aab^ | 0.84± 0.07^AB*^ | 0.78± 0.07^AB*^ | 0.61± 0.05^B*^ | 0.71± 0.06^B^ | 1.20± 0.06^a*^ | 1.35± 0.09^a*^ | 0.92± 0.08^bc*^ | 0.83± 0.07^c^ |  |
| ***bcl2-like*** | 1.02±0.06^a^ | 1.02± 0.10^*^ | 0.82± 0.05^*^ | 0.79± 0.03 | 0.86± 0.05 | 0.72± 0.04^bc*^ | 0.58± 0.05^c*^ | 0.68± 0.05^bc^ | 0.81± 0.06^ab^ |  |
| ***il-6*** | 1.06±0.12^Bb^ | 2.22± 0.25^A^ | 0.72± 0.04^B^ | 1.01± 0.10^B^ | 0.76± 0.06^B^ | 1.80± 0.22^a^ | 0.85± 0.14^b^ | 0.82± 0.07^b^ | 0.99± 0.18^b^ |  |
| ***tnfα*** | 1.11±0.16^AB^ | 1.47± 0.21^AB^ | 0.94± 0.14^B*^ | 1.52± 0.16^A^ | 1.21± 0.10^AB^ | 1.49± 0.18 | 1.69± 0.18^*^ | 1.47± 0.16 | 1.87± 0.44 |  |

**Table 7:** Quantitative expression of *tlr2*, *tlr9*, *nod1*, *nod2*, *nf-κB*, *stat3*, *bcl2-like*, *il-6* and *tnfα* for posterior-intestine of European sea bass (*Dicentrarchus labrax*) after bacterial bath-challenge with 5 x 10^5^ CFU mL^-1^ *T. maritimum*. Data are expressed as mean ± SEM (n=12 per treatment). Different capital letters in the same row stand for differences between control and mock-challenge and lower case letters indicate significant differences between control and challenged groups, while (*) represents statistical differences between mock and challenged fish at each sampling point (One-way ANOVA or Kruskal-Wallis; p≤0.05).

| **Genes** | **Control** | **Mock-challenged** | | | | **Challenged** | | | | |
| --- | --- | --- | --- | --- | --- | --- | --- | --- | --- | --- |
|  | **0 h** | **6 h** | **24 h** | **48 h** | **72 h** | **6 h** | **24 h** | **48 h** | **72 h** |  |
| ***tlr2*** | 1.01±0.05^ab^ | 1.07±0.06^*^ | 1.17±0.07^*^ | 1.17±0.09 | 1.22±0.08 | 0.81±0.07^bc*^ | 0.68±0.08^c*^ | 0.99±0.12^ab^ | 1.09±0.09^a^ |  |
| ***tlr9*** | 1.02±0.06^B^ | 1.55±0.11^A*^ | 1.57±0.10^A*^ | 1.38±0.19^AB^ | 1.43±0.13^A^ | 1.07±0.12^*^ | 0.94±0.12^*^ | 1.01±0.11 | 1.38±0.15 |  |
| ***nod1*** | 1.04±0.09 | 0.91±0.05 | 0.99±0.06 | 1.07±0.07 | 1.21±0.12 | 1.00±0.08 | 1.40±0.23 | 1.01±0.05 | 1.03±0.09 |  |
| ***nod2*** | 1.05±0.10^B^ | 1.59±0.18^AB*^ | 1.91±0.17^A*^ | 1.07±0.11^B^ | 1.51±0.18^AB^ | 0.86±0.12^*^ | 1.41±0.46^*^ | 1.04±0.13 | 1.44±0.14 |  |
| ***nf-κB*** | 1.05±0.10^Aa^ | 0.74±0.05^B*^ | 0.64±0.04^B*^ | 0.59±0.05^B^ | 0.60±0.03^B^ | 1.07±0.10^a*^ | 1.04±0.14^a*^ | 0.60±0.06^b^ | 0.63±0.03^b^ |  |
| ***stat3*** | 1.02±0.07 | 0.87±0.05^*^ | 0.86±0.04^*^ | 1.01±0.09 | 0.88±0.06 | 1.12±0.06^*^ | 1.19±0.11^*^ | 0.92±0.06 | 0.88±0.06 |  |
| ***bcl2-like*** | 1.03±0.07^Aa^ | 0.72±0.05^B^ | 0.88±0.05^AB*^ | 0.73±0.05^B^ | 0.74±0.04^B^ | 0.79±0.06^b^ | 0.69±0.05^b*^ | 0.69±0.04^b^ | 0.68±0.05^b^ |  |
| ***il-6*** | 1.06±0.12 | 1.03±0.10 | 1.02±0.10 | 0.98±0.09^*^ | 0.89±0.07 | 0.91±0.10 | 1.13±0.15 | 0.75±0.05^*^ | 0.88±0.08 |  |
| ***tnfα*** | 1.06±0.11^a^ | 1.15±0.20 | 1.13±0.10^*^ | 1.46±0.19^*^ | 1.03±0.08 | 1.16±0.17^a^ | 0.65±0.13b^*^ | 0.86±0.12^ab*^ | 1.30±0.18^a^ |  |
